# Community risks for SARS-CoV-2 infection among fully vaccinated US adults by rurality: A retrospective cohort study from the National COVID Cohort Collaborative

**DOI:** 10.1371/journal.pone.0279968

**Published:** 2023-01-05

**Authors:** Alfred Jerrod Anzalone, Jing Sun, Amanda J. Vinson, William H. Beasley, William B. Hillegass, Kimberly Murray, Brian M. Hendricks, Melissa Haendel, Carol Reynolds Geary, Kristina L. Bailey, Corrine K. Hanson, Lucio Miele, Ronald Horswell, Julie A. McMurry, J. Zachary Porterfield, Michael T. Vest, H. Timothy Bunnell, Jeremy R. Harper, Bradley S. Price, Susan L. Santangelo, Clifford J. Rosen, James C. McClay, Sally L. Hodder, on behalf of the National COVID Cohort Collaborative (N3C) Consortium

**Affiliations:** 1 University of Nebraska Medical Center, Omaha, Nebraska, United States of America; 2 Johns Hopkins University, Baltimore, Maryland, United States of America; 3 Nova Scotia Health Authority, Halifax, Nova Scotia, Canada; 4 University of Oklahoma, Norman, Oklahoma, United States of America; 5 University of Mississippi Medical Center, Jackson, Mississippi, United States of America; 6 Maine Health Institute for Research, Portland, Maine, United States of America; 7 West Virginia University, Morgantown, West Virginia, United States of America; 8 University of Colorado Anschutz Medical School, Aurora, CO, United States of America; 9 Louisiana State University Health Sciences Center, New Orleans, Louisiana, United States of America; 10 Oregon State University, Corvallis, Oregon, United States of America; 11 University of Kentucky, Lexington, Kentucky, United States of America; 12 Christiana Care Health System, Newark, Delaware, United States of America; 13 Nemours Children’s Health, Wilmington, Delaware, United States of America; 14 Owl Health Networks, Indianapolis, Indiana, United States of America; 15 Tufts University School of Medicine, Boston, Massachusetts, United States of America; University of New Mexico Health Sciences Center, UNITED STATES

## Abstract

**Background:**

While COVID-19 vaccines reduce adverse outcomes, post-vaccination SARS-CoV-2 infection remains problematic. We sought to identify community factors impacting risk for breakthrough infections (BTI) among fully vaccinated persons by rurality.

**Methods:**

We conducted a retrospective cohort study of US adults sampled between January 1 and December 20, 2021, from the National COVID Cohort Collaborative (N3C). Using Kaplan-Meier and Cox-Proportional Hazards models adjusted for demographic differences and comorbid conditions, we assessed impact of rurality, county vaccine hesitancy, and county vaccination rates on risk of BTI over 180 days following two mRNA COVID-19 vaccinations between January 1 and September 21, 2021. Additionally, Cox Proportional Hazards models assessed the risk of infection among adults without documented vaccinations. We secondarily assessed the odds of hospitalization and adverse COVID-19 events based on vaccination status using multivariable logistic regression during the study period.

**Results:**

Our study population included 566,128 vaccinated and 1,724,546 adults without documented vaccination. Among vaccinated persons, rurality was associated with an increased risk of BTI (adjusted hazard ratio [aHR] 1.53, 95% confidence interval [CI] 1.42–1.64, for urban-adjacent rural and 1.65, 1.42–1.91, for nonurban-adjacent rural) compared to urban dwellers. Compared to low vaccine-hesitant counties, higher risks of BTI were associated with medium (1.07, 1.02–1.12) and high (1.33, 1.23–1.43) vaccine-hesitant counties. Compared to counties with high vaccination rates, a higher risk of BTI was associated with dwelling in counties with low vaccination rates (1.34, 1.27–1.43) but not medium vaccination rates (1.00, 0.95–1.07). Community factors were also associated with higher odds of SARS-CoV-2 infection among persons without a documented vaccination. Vaccinated persons with SARS-CoV-2 infection during the study period had significantly lower odds of hospitalization and adverse events across all geographic areas and community exposures.

**Conclusions:**

Our findings suggest that community factors are associated with an increased risk of BTI, particularly in rural areas and counties with high vaccine hesitancy. Communities, such as those in rural and disproportionately vaccine hesitant areas, and certain groups at high risk for adverse breakthrough events, including immunosuppressed/compromised persons, should continue to receive public health focus, targeted interventions, and consistent guidance to help manage community spread as vaccination protection wanes.

## Introduction

The recent surge in SARS-CoV-2 cases associated with the Delta and Omicron variants [1] highlights the potential COVID-19 risk for unvaccinated and vaccinated persons across the United States (US). Recent studies demonstrate increased risk over time in those with prior SARS-CoV-2 infection [2], among fully vaccinated healthcare workers with known risk factors [3], within households with prolonged exposure [4], and in specific immunosuppressed populations [5]. Numerous test-negative case-control design studies demonstrate vaccine effectiveness in reducing severe COVID-19 outcomes [6, 7], including a potential reduction of COVID-19 deaths through May 2021 in the US [8], and symptomatic COVID-19 [9], but understanding the role of community susceptibility is crucial to controlling SARS-CoV-2 and reducing spread among those who may have an inadequate response to vaccination due to immune status.

Vaccines save millions of lives annually and protect against more than 20 diseases [10], yet no vaccine is entirely effective. Despite a widespread understanding of the importance of vaccination in combatting disease, vaccine hesitancy has been on the rise for decades [11] particularly in high-income countries [12]. Few patient-level studies consider the impact of community factors on an individual’s risk for SARS-CoV-2 infection. Previous work has identified an increased risk of COVID-19 related deaths and hospitalization among rural-dwellers compared to urban-dwellers [13] and associations between immune suppression and risk of post-vaccination SARS-CoV-2 infections [5]. However, to our knowledge, no large-scale, multi-site study has investigated differences in breakthrough infections (BTI) based on community risk profiles. Racial/ethnic differences [14], rurality [15], political affiliation [16], and other factors impact community vaccination rates. This study seeks to explore associations between community factors–notably rurality, community vaccination rates, and community vaccine hesitancy–and SARS-CoV-2 BTI in a large US sample.

## Methods

This retrospective cohort study received Institutional Review Board (IRB) approval from the University of Nebraska Medical Center (0176-21-EP) and Johns Hopkins University (IRB00309495). The N3C Data Access Committee approved this study, which operates under the authority of the National Institutes of Health IRB with Johns Hopkins University School of Medicine serving as the central IRB. No informed consent was obtained because the study used a limited data set.

Our study cohort includes persons receiving two documented doses of a messenger RNA (mRNA) vaccine (BNT162b2 or mRNA-1273) between January 1, 2021, and September 21, 2021. Person-time at risk for BTI accrued for all vaccinated persons from the date of second COVID-19 vaccination until the earliest: 1) BTI, 2) death, 3) transfer to hospice, 4) third dose of COVID-19 vaccination, 5) December 20, 2021 (end of ascertainment period), or 6) 180 days. We did not include persons vaccinated in December 2020 because vaccination rollout in the US began with frontline healthcare workers, and they may have had higher exposure risks than the general population.

Also included are persons unvaccinated or with undocumented vaccination status (UUVS) AND at least one provider visit (outpatient, ED, inpatient) between January 1, 2021, and September 21, 2021 to compare baseline risk in a comparison population of persons with health system interaction during the study period. Person-time at risk is accrued for UUVS persons based on the first visit (outpatient, emergency, observation stay, or inpatient) with each center in 2021 for SARS-CoV-2 infection during the study period until the earliest: 1) SARS-CoV-2 infection, 2) death, 3) transfer to hospice, 4) December 20, 2021 (end of ascertainment period), or 5) 180 days.

This study followed the Enhancing the Quality and Transparency of Health Research (EQUATOR) reporting guidelines: Reporting of Studies Conducted Using Observational Routinely Collected Health Data (RECORD) [17]. Data extraction and analyses were performed using PySpark, SQL, and R version 3.5.1. within the N3C Enclave in accordance with N3C privacy [18] and download review policies.

### N3C Data Enclave

The N3C Enclave has broad inclusion criteria, harmonizing data from 72 sites across the US [18]. N3C collects longitudinal Electronic Health Record (EHR) or Health Information Exchange (HIE) data (with a lookback period to January 2018) on all persons with a positive SARS-CoV-2 polymerase chain reaction (PCR), antigen, or antibody test or a COVID-19 diagnostic code without a confirmed positive diagnostic test. N3C includes a demographically matched comparison group of SARS-CoV-2 uninfected [19]. Source system SARS-CoV-2 testing protocols are mapped to standard terminologies for labs (LOINC) and COVID-19 conditions (ICD-10 CM and SNOMED CT) by the N3C Data Ingestion and Harmonization Workstream, which maintains a computable phenotype for defining presence of COVID-19 [20]. S1 Methods in S1 File provides an overview of the ingestion and harmonization process, sampling approaches, and overall structure of the N3C Enclave, concept set definitions, and computable phenotypes utilized.

### Data extraction

Data were extracted on March 24, 2022 (N3C release 71), in the OMOP Common Data Model version 5.3.1 [21]. This facilitates a minimum of 90 days for data reporting from second vaccine administration event cutoff (September 21, 2021) through our minimum data partner reporting period (December 20, 2021). All clinical concept sets were created collaboratively within the N3C Enclave, with at least one informatician and one clinical subject-matter expert reviewing each relevant concept set. Concept sets contain standardized terminology corresponding to clinical domains (e.g., LOINC, SNOMED CT, ICD-10-CM, RxNorm).

We included persons in this study based on N3C data partner reporting practices. Our primary data partner requirements were vaccination reporting and 5-digit ZIP Code availability. S1 Fig in S1 File provides a profile of data partners included in this study, S2 Fig in S1 File reports vaccine administration and visit availability at the site level, and S1 Methods in S1 File details our sampling approach. Our study cohort includes adults (≥18 years) who received two mRNA COVID-19 vaccinations between January 1, 2021, and September 21, 2021. Persons with missing age or gender were excluded. Also excluded were persons with a primary vaccination of adenovirus vector vaccine (Ad26.COV2.S [Johnson and Johnson]) due to increasing evidence of decreased efficacy [22], waning immunity [23], and previous work published in N3C [5] showing reduced efficacy compared to mRNA vaccines.

### Definition of key factors

The analytical dataset included the two mRNA COVID-19 vaccines currently given US Food and Drug Administration (FDA) authorization: BNT162b2 (Pfizer–BioNTech) and mRNA‐1273 (Moderna). The primary outcome in this study is BTI following a second mRNA COVID-19 vaccination administration event (≥14 days following vaccination). We define COVID-19 positivity and timing by considering breakthrough cases with a definitive polymerase chain reaction (PCR) or SARS-CoV-2 Antigen test following primary vaccination (i.e., second mRNA dose).

Persons were classified based on rurality, vaccine hesitancy, and county vaccination rate through September 21, 2021. Rurality was identified by mapping 5-digit ZIP Codes to the 2010 Rural-Urban Continuum Codes (RUCA) [24]. We grouped RUCA codes into a binary urban-rural distinction and further, subdivided by degree of rurality, using a previously validated methodology, into urban, urban-adjacent rural (UAR), and nonurban-adjacent rural (NAR) for modeling [13]. To capture population-level vaccine hesitancy, we utilized data from the COVID-19 Trends and Impact Survey [25], which is the largest US public health survey (including more than 5 million responses targeting vaccination hesitancy between January and May 2021) that has operating continuously to gather public trends and public perception on COVID-19 since April 2020, through October 1, 2021. This survey includes in-depth geographic resolution and has included questions on vaccination perception and uptake at the ZIP Code level collapsed into a single weighted score to establish changes in vaccine hesitancy by region over time. We categorized vaccine hesitancy into three groups based on patient distribution (interquartile ranges) in our sample: low (≤5% county hesitancy), medium (6–15% county hesitancy), and high (>15% county hesitancy). County vaccination rates were mapped from counties using the U.S. Department of Housing and Urban Development (HUD) crosswalk [26] to patient ZIP Code for adult county vaccination status reported by the US Centers for Disease Control and Prevention (CDC) through September 21, 2021 [27]. We categorized vaccination rates into three groups based on patient distribution (interquartile ranges) in our sample: low (<70% county vaccination), medium (70–77% county vaccination), and high (>77% county vaccination).

### Covariates

We utilized demographics (age, sex, and race/ethnicity) and diagnoses of comorbid conditions from 1/1/2018 until the date of the second vaccination event or through the censor date in patients with undocumented vaccination status. We assessed for the impact of comorbid conditions using eight collapsed classes from the Quan-Charlson Comorbidity Index [28], documented obesity (using measurement data and condition diagnoses, preferentially in that order), and immunosuppressed/compromised status defined as those with any of the following conditions: solid organ transplant (SOT), bone marrow transplant (BMT), autoimmune rheumatic disease (RD), multiple sclerosis (MS), or human immunodeficiency virus (HIV). We classified geographic regions based on patient’s residential state or ZIP Code into four Census-derived categories: northeast, midwest, west, or south. To account for baseline risk of BTI changing with the predominance of the Delta variant in the U.S., we used June 20, 2021, to stratify our analyses into pre- and post-Delta periods based on the U.S. CDC reporting Delta as the dominant U.S. variant [1]. Because our study period ends before the predominance of the Omicron variant and adequate follow-up time is not available, Omicron is excluded from this stratification. Covariates were selected based on *a priori* knowledge of community risk [29], patient-level vulnerability [5, 30], and data availability [19].

### Statistical analyses

Summary statistics using Pearson’s Chi-squared test and Wilcoxon rank sum tests were calculated on all subjects stratified by rurality. We used multivariable Cox proportional hazards models to assess time to BTI following two mRNA vaccination events. Cox proportional hazards models were assessed on key factors individually and combined. Time to BTI was assessed within 180 days of vaccination. Patient time was censored at: 1) breakthrough event, 2) third vaccine administration, 3) death or transfer to hospice, 4) end of risk period (180 days), or 5) end of study period or latest data partner reporting date. We used Kaplan-Meier cumulative incidence curves to demonstrate time to BTI by rurality, vaccine hesitancy, and county vaccination rates. Incidence curves were compared with a log-rank test.

To provide a risk comparison for disease transmission in the absence of vaccination, we evaluated, using Cox proportional hazard models, risk of SARS-CoV-2 infection over the same study period in subjects (from the same data providers) who were either unvaccinated or with undocumentated vaccination status. To assess for differences in disease severity following vaccination, we compared differences in post-COVID outcomes in the vaccinated and UUVS cohorts using multivariable logistic regression modeling for hospitalization and adverse events (mechanical ventilation or death) within 30 days of SARS-CoV-2 infection.

Sensitivity analyses included: assessing the potential interaction between rurality, vaccine hesitancy, and county vaccination rates; including and excluding those with previous SARS-CoV-2 infection; varying the time of breakthrough definitions (+/- 7 days); and assessing for differences in data partner reporting practices using mixed-effects modeling with data contributing partner as a covariate in the model to determine if observed point estimates are artifacts of these differences or represent a referral-in bias with sites some sites seeing a disproportionate number of persons from underserved communities. We also ran a sensitivity analysis using the CDC-developed Social Vulnerability Index (SVI) [31], which combines 15 US Census variables into an aggregate measure to determine county disaster preparedness. We did so to assess sensitivity to a community measure aggregated around county preparedness (socioeconomic status, household composition, housing/transportation, and race/ethnicity/language) rather than those selected for this study, which were done so to assess susceptibility to SARS-CoV-2 that may largely be attributed to differences in education, attitudes, and public health reach. A strong interaction was observed between rurality and vaccine hesitancy for BTI (*p* for interaction < 0.001). Based on this observed interaction, adjusted analyses were secondarily stratified by rurality, which facilitates both between- and within-strata comparisons to assess independent associations between vaccine hesitancy and dwelling status.

All statistical analysis were performed in R v3.5.1. Survival analysis was performed using the ‘survival’ [32], ‘coxme’ [33], and ‘car’ [34] packages. Base ‘stats’ package was used to perform Pearson’s Chi-squared, analysis of variance (ANOVA), and Wilcoxon rank sum tests. Data visualization was performed using the ‘ggplot2’ [35] package. P-values < 0.05 were considered statistically significant in hypothesis. All p-values presented are for 2-sided tests.

## Results

### Demographic and clinical characteristics

**Fig 1:**
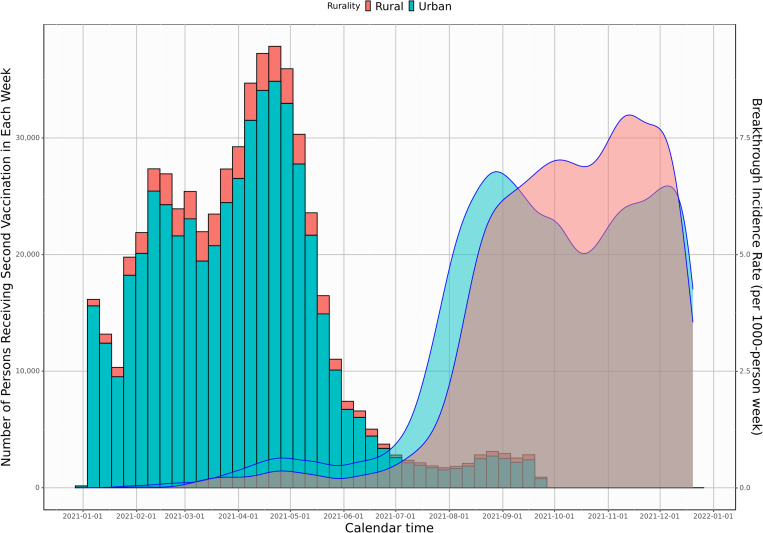
Number vaccination and breakthrough infection density by week by rurality, January 1, 2021—December 20, 2021. ^a^Bars represent number vaccinated and lines represent breakthrough infection rates.

Our sample included 567,041 vaccinated subjects and 1,724,546 UUVS subjects over the study period (S3 Fig in S1 File). Most patients received two doses in April through May 2021 (Fig 1). Most breakthrough events happened after August 2021, with an earlier spike in urban dwellers and a later, more prominent spike in October and December 2021 in rural dwellers. The vaccinated sample (Table 1) included a median (interquartile range [IQR]) age of 52 (36–67), 331,004 female (58%), and 236,037 male (42%). Rural dwellers had similar gender distribution but were older and less racially and ethnically diverse than urban dwellers. Urban dwellers had higher comorbid burden (*p*<0.001) for liver disease while rural dwellers had higher comorbid burden (*p*<0.001) for peripheral vascular disease, peptic ulcer disease, diabetes, stroke, renal disease, cancer, and immunocompromised/suppressed conditions (solid organ transplant, multiple sclerosis, autoimmune rheumatic disease, bone marrow transplant, and HIV). Rural dwellers had higher incidence of obesity (42%) than urban dwellers (34%). Rural dwellers were more likely to live in a vaccine-hesitant county (median [IQR] hesitancy 13% [10–18%] vs. urban 7% [5–10%]) and have lower county vaccination rates (64% [59–69%] vs. urban 74% [70–78%]).

**Table 1:** Baseline characteristics of all patients in N3C receiving 2 doses of mRNA vaccine between January 1, 2021, and September 21, 2021.

Characteristic	Overall, N = 567,041^1^	Rural Categorization^2^			
		Urban, N = 516,362^1^	Urban-Adjacent Rural, N = 42,399^1^	Nonurban-Adjacent Rural, N = 8,280^1^	*P* value^2^
Number of SARS-CoV-2 Breakthrough Cases	8,369 (1.5%)	7,267 (1.4%)	913 (2.2%)	189 (2.3%)	<0.001
Age, Median (IQR)	52 (36, 67)	52 (36, 66)	58 (41, 71)	61 (45, 72)	<0.001
Age Category					<0.001
<30	82,449 (15%)	77,025 (15%)	4,686 (11%)	738 (8.9%)	
30–49	173,103 (31%)	160,615 (31%)	10,671 (25%)	1,817 (22%)	
50–64	145,196 (26%)	131,924 (26%)	10,932 (26%)	2,340 (28%)	
65–75	104,593 (18%)	93,800 (18%)	8,880 (21%)	1,913 (23%)	
>75	61,700 (11%)	52,998 (10%)	7,230 (17%)	1,472 (18%)	
Gender					0.004
Female	331,004 (58%)	301,773 (58%)	24,441 (58%)	4,790 (58%)	
Male	236,037 (42%)	214,589 (42%)	17,958 (42%)	3,490 (42%)	
Race/Ethnicity					<0.001
Non-Hispanic/Latinx White	347,622 (61%)	305,994 (59%)	34,526 (81%)	7,102 (86%)	
Non-Hispanic/Latinx Black	54,455 (9.6%)	51,980 (10%)	2,130 (5.0%)	345 (4.2%)	
Hispanic/Latinx	79,010 (14%)	74,987 (15%)	3,503 (8.3%)	520 (6.3%)	
Asian American/Pacific Islander	28,301 (5.0%)	27,721 (5.4%)	547 (1.3%)	33 (0.4%)	
Other	42,240 (7.4%)	40,822 (7.9%)	1,239 (2.9%)	179 (2.2%)	
Missing/Unknown	15,413 (2.7%)	14,858 (2.9%)	454 (1.1%)	101 (1.2%)	
Vaccine Hesitancy, Median (IQR)	0.08 (0.05, 0.11)	0.07 (0.05, 0.10)	0.13 (0.10, 0.17)	0.16 (0.10, 0.21)	<0.001
Vaccine Hesitancy Category ^3^					<0.001
Low (≤ 5% hesitant)	193,054 (34%)	187,597 (36%)	4,889 (12%)	568 (6.9%)	
Medium (6–15% hesitant)	323,266 (57%)	298,738 (58%)	21,182 (50%)	3,346 (40%)	
High (>15% hesitant)	50,721 (8.9%)	30,027 (5.8%)	16,328 (39%)	4,366 (53%)	
County Vaccination Rate ^3^	74 (69, 77)	74 (70, 78)	65 (60, 69)	63 (55, 68)	<0.001
County Vaccination Rate Category ^3^					<0.001
Low (<70%)	187,702 (33%)	148,998 (29%)	32,030 (76%)	6,674 (81%)	
Medium (70–77%)	244,186 (43%)	237,049 (46%)	6,524 (15%)	613 (7.4%)	
High (>77%)	135,153 (24%)	130,315 (25%)	3,845 (9.1%)	993 (12%)	
U.S. Census Region					<0.001
Northeast	95,154 (17%)	94,911 (18%)	152 (0.4%)	91 (1.1%)	
Midwest	154,785 (27%)	128,213 (25%)	21,712 (51%)	4,860 (59%)	
South	97,132 (17%)	88,551 (17%)	7,383 (17%)	1,198 (14%)	
West	219,970 (39%)	204,687 (40%)	13,152 (31%)	2,131 (26%)	
Charlson Comorbidity Index, Median (IQR)	1 (0, 2)	1 (0, 2)	1 (0, 3)	1 (0, 3)	<0.001
Number of Comorbid Conditions					<0.001
0 Comorbid Conditions	289,877 (51%)	267,045 (52%)	19,219 (45%)	3,613 (44%)	
1 Comorbid Conditions	131,930 (23%)	119,695 (23%)	10,211 (24%)	2,024 (24%)	
2 Comorbid Conditions	66,341 (12%)	59,635 (12%)	5,609 (13%)	1,097 (13%)	
> = 3 Comorbid Conditions	78,893 (14%)	69,987 (14%)	7,360 (17%)	1,546 (19%)	
Comorbid Conditions ^3^					
Heart Disease	54,404 (9.6%)	47,781 (9.3%)	5,413 (13%)	1,210 (15%)	<0.001
Peripheral Vascular Disease	59,148 (10%)	52,801 (10%)	5,252 (12%)	1,095 (13%)	<0.001
Peptic Ulcer Disease	11,041 (1.9%)	9,885 (1.9%)	976 (2.3%)	180 (2.2%)	<0.001
Mild or Severe Liver Disease	46,345 (8.2%)	42,617 (8.3%)	3,162 (7.5%)	566 (6.8%)	<0.001
Diabetes Mellitus	112,188 (20%)	100,810 (20%)	9,448 (22%)	1,930 (23%)	<0.001
Hemiplegia or Paraplegia	5,880 (1.0%)	5,333 (1.0%)	467 (1.1%)	80 (1.0%)	0.3
Stroke	45,844 (8.1%)	40,813 (7.9%)	4,212 (9.9%)	819 (9.9%)	<0.001
Renal Disease	52,426 (9.2%)	45,709 (8.9%)	5,535 (13%)	1,182 (14%)	<0.001
Any Cancer (Except Skin)	66,934 (12%)	60,027 (12%)	5,738 (14%)	1,169 (14%)	<0.001
Documented Obesity Before Vaccination	197,716 (35%)	176,293 (34%)	17,697 (42%)	3,726 (45%)	<0.001
ISC Status (RD, SOT, MS, HIV, BMT)	68,890 (12%)	61,810 (12%)	5,876 (14%)	1,204 (15%)	<0.001
Vaccine Manufacturer					<0.001
Pfizer BioNTech	413,152 (73%)	378,455 (73%)	28,992 (68%)	5,705 (69%)	
Moderna NIAID	153,889 (27%)	137,907 (27%)	13,407 (32%)	2,575 (31%)	
Month of Full Vaccination					<0.001
January 2021	59,588 (11%)	55,889 (11%)	3,081 (7.3%)	618 (7.5%)	
February 2021	100,086 (18%)	91,415 (18%)	7,146 (17%)	1,525 (18%)	
March 2021	111,932 (20%)	100,012 (19%)	9,839 (23%)	2,081 (25%)	
April 2021	156,173 (28%)	142,671 (28%)	11,375 (27%)	2,127 (26%)	
May 2021	86,679 (15%)	79,577 (15%)	6,058 (14%)	1,044 (13%)	
June 2021	23,872 (4.2%)	21,515 (4.2%)	2,019 (4.8%)	338 (4.1%)	
July 2021	9,550 (1.7%)	8,665 (1.7%)	761 (1.8%)	124 (1.5%)	
August 2021	10,935 (1.9%)	9,638 (1.9%)	1,084 (2.6%)	213 (2.6%)	
September 2021 (through 9/21/21)	8,226 (1.5%)	6,980 (1.4%)	1,036 (2.4%)	210 (2.5%)	

1. Statistics presented: n (%), Median (Interquartile Range [IQR])

2. Wilcoxon Rank Sum Test; Pearson’s Chi Squared Test

3. Definitions and logic used for rural categorization, vaccine hesitancy, county vaccination, and comorbid conditions provided in **eMethods 1**

The UUVS sample included a median (IQR) age of 46 (32–61), 991,990 female (58%), and 732,556 male (42%). Patients without documented vaccinations had similar demographic characteristics (S1 Table in S1 File) except the overall comorbid burden was lower than in the vaccinated cohort. UUVS lived in counties with similar, albeit lower, median adult vaccination rates (71% [63–75%]) compared to vaccinated patients (74% [69–77%]). UUVS had 343,348 new SARS-CoV-2 infections during the study period (20%) compared to 8,369 new SARS-CoV-2 infections in the vaccinated cohort (1.5%). A significantly larger percentage of UUVS (17%) lived in rural rather than urban areas in the vaccinated (8.9%) study sample. Among those dwelling in rural areas, a larger proportion lived in high vaccine hesitant counties in the UUVS (55%) compared to the vaccinated (41%) cohort.

### Kaplan-Meier cumulative incidence curves in the vaccinated cohort

**Fig 2:**
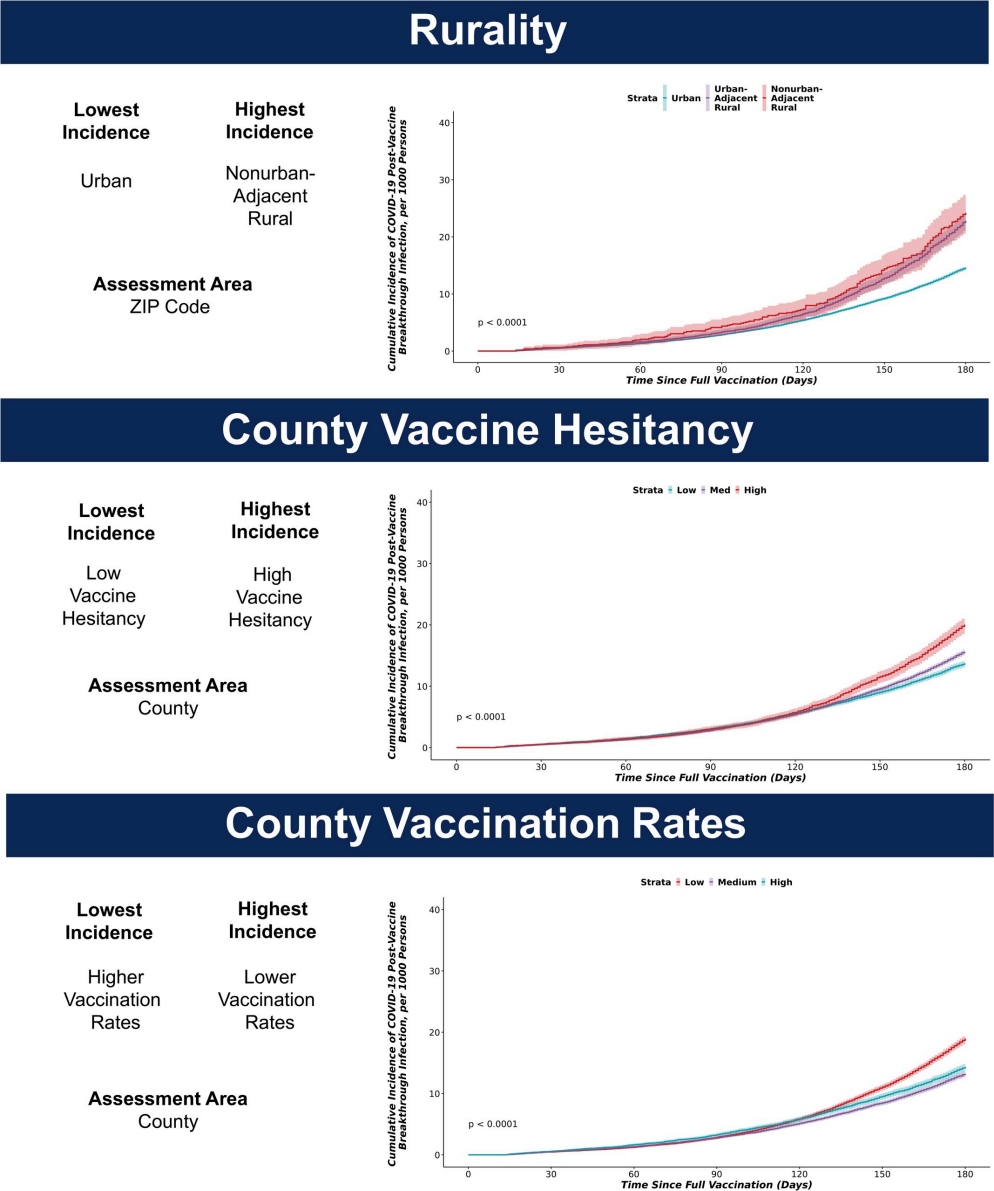
Kaplan Meier cumulative incidence curves for SARS-CoV-2 breakthrough infection following two mRNA vaccinations in adults, January 1, 2021 –December 20, 2021.

Kaplan Meier cumulative incidence curves demonstrated significantly higher cumulative incidence of BTI in three key areas shown in Fig 2 and S4 Fig in S1 File. The highest cumulative incidence was observed in UAR and NAR vs urban areas (p<0.001), high vaccine hesitancy vs medium and low vaccine hesitancy (p<0.001), and low vaccination rates vs. medium and high vaccination rates (p<0.001).

### Multivariable-adjusted hazard ratios for breakthrough SARS-CoV-2 infection by community vulnerability in the vaccinated cohort

**Fig 3:**
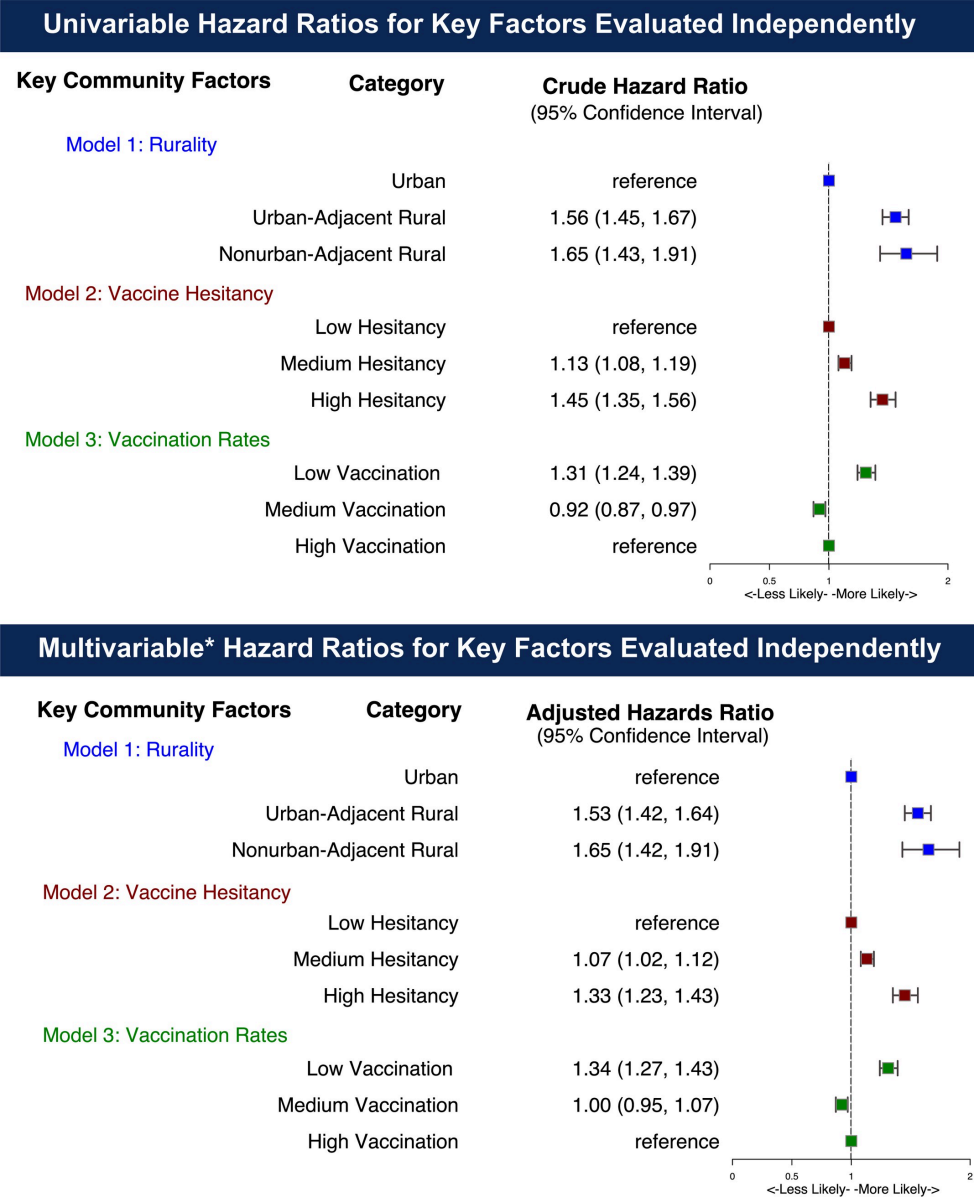
Univariable and multivariable Cox-proportional hazard ratios for SARS-CoV-2 breakthrough infection following two mRNA vaccinations by community factors, January 1, 2021 –December 20, 2021. A. Univariable Hazard Ratios for Key Factors Evaluated Independently. B. Multivariable* Hazard Ratios for Key Factors Evaluated Independently. *Models adjusted for age, gender, race/ethnicity, comorbid conditions, prior SARS-CoV-2 infection, vaccine manufacturer, period of vaccination (relative to Delta dominance), and US Census region. Full model specifications available in S2 and S3 Tables in S1 File.

There was an increased hazard ratio across key community-based exposures in vaccinated individuals (Fig 3) in both univariable and multivariable analyses assessed independently. Univariable hazard ratios demonstrated higher risk of BTI in persons dwelling in: 1) rural areas (hazard ratio [HR] 1.56 [95% confidence interval 1.45–1.67] for UAR and 1.65 [1.43–1.91] for NAR relative to urban dwellers), 2) counties with higher vaccine hesitancy (1.13 [1.08–1.19] for medium and 1.45 [1.35–1.56] for high relative to low vaccine hesitancy), and 3) counties with lower vaccination rates (1.31 [1.24–1.39] for low relative to high vaccination rates). After adjusting for age, gender, race/ethnicity, obesity, comorbid conditions, time period (relative to Delta dominance), prior COVID-19 infection before vaccination, and US Census regions, higher risk of BTI was observed in persons dwelling in: 1) rural areas (1.53 [1.42–1.64] for UAR and 1.65 [1.42–1.91] NAR relative to urban dwellers), 2) counties with higher vaccine hesitancy (1.07 [1.02–1.12] for medium and 1.33 [1.23–1.43] for high relative to low vaccine hesitancy), and 3) counties with lower vaccination rates (1.34 [1.27–1.43] for low relative to high vaccination rates). Univariable and multivariable hazard ratios for all model covariates are available in S2 and S3 Tables in S1 File, respectively.

**Fig 4:**
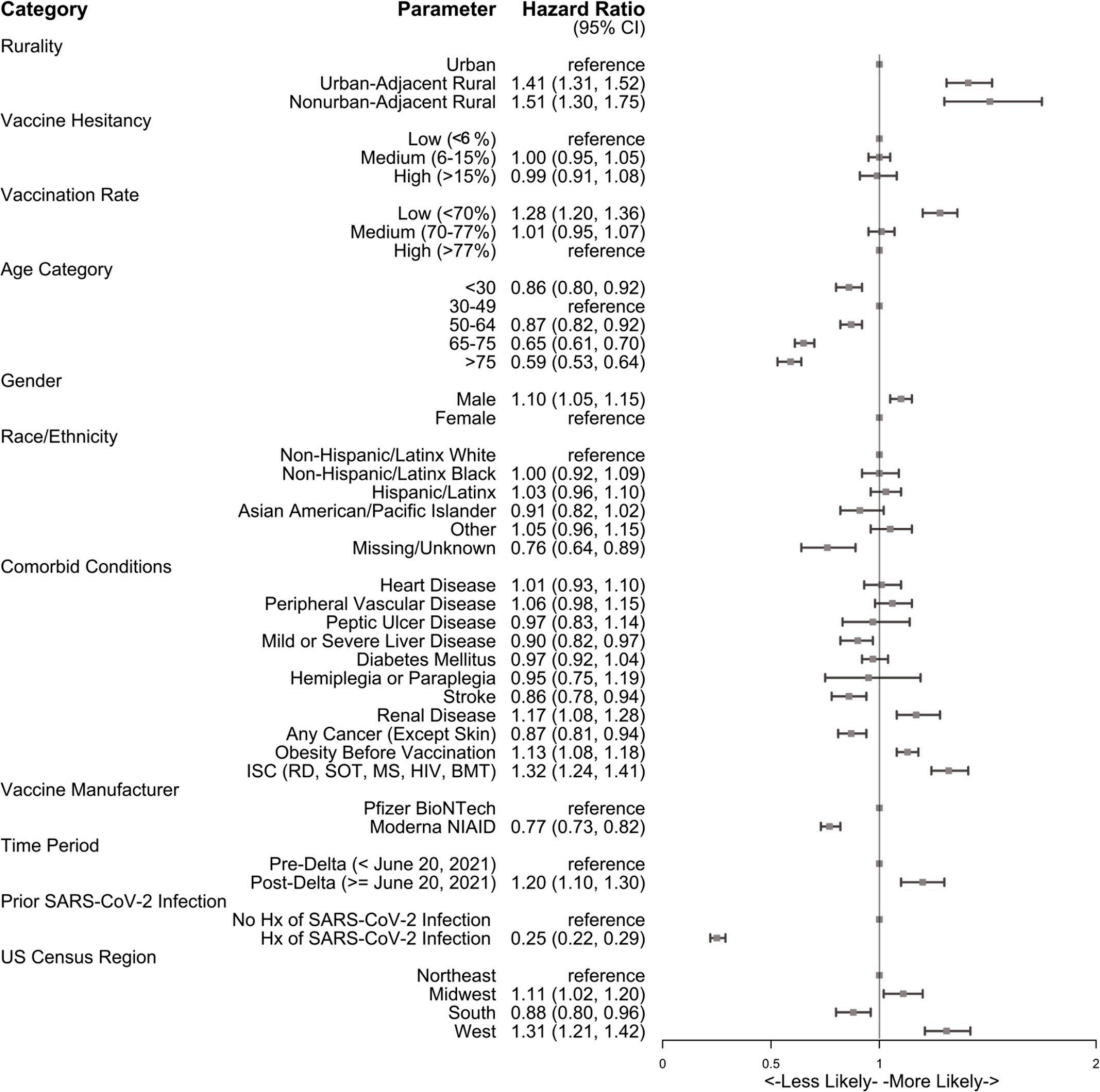
Multivariable Cox-proportional hazard ratios for SARS-CoV-2 breakthrough infection following two mRNA vaccinations in adults by combined key factors, January 1, 2021 –December 20, 2021.

As shown in Fig 4, combining key community factors into a single adjusted model attenuated some of the risk across all community factors. After adjusting for demographic differences and comorbid burden, rurality (1.41 [1.31–1.52] for UAR and 1.51 [1.30–1.75] NAR relative to urban dwellers) and county vaccination rates (1.28 [1.20–1.36] for low relative to high county vaccination rates) were associated with increased adjusted risk for breakthrough infection. Pre-vaccination SARS-CoV-2 infection (0.25 [0.22–0.29]) and receiving a primary vaccination of the Moderna vaccine (0.77 [0.73–0.82] relative to Pfizer) reduced risk of BTI during the study period. Limited difference was observed in region, race/ethnicity, and gender, but age was inversely correlated with BTI. Immunocompromised/suppressed conditions (1.32 [1.24–1.41]), obesity (1.13 [1.08–1.18]), and post-Delta vaccination (1.20 [1.10–1.30]) were associated with an increased risk of BTI.

Sensitivity analyses for interaction between vaccine hesitancy and rurality showed significant interaction terms. To account for this, we stratified risk of BTI by binary rurality (UAR and NAR combined due to small sample sizes in NAR at stratification level), which demonstrated variance among other key factors independent of dwelling status (S4 Table in S1 File). While there was no statistically significant difference between vaccine hesitancy and vaccination rates in rural dwellers, urban dwellers had higher risk associated with lower vaccination rates. We also assessed for differences based on categorical level for vaccine hesitancy, removing patients with prior SARS-CoV-2 infection, and including data partner as a random effect to assess for differences in data partner reporting and dwelling heterogeneity (S5 Table in S1 File). To determine if observed point estimates were inflated due to sample size or potential overrepresentation in study sites, we assessed sensitivity to an additional community measure by running the same analyses with SVI. This county estimate showed no increased risk of BTI in the study cohort, suggesting that not all community factors are associated with an increased risk of BTI. Overall, sensitivity analyses demonstrated similar findings to those in primary analyses.

### Multivariable-adjusted hazard ratios for SARS-CoV-2 infection by community vulnerability in UUVS cohort

After adjusting for age, gender, race/ethnicity, obesity, comorbid conditions, prior SARS-CoV-2 infection, and US Census regions, there was an increased hazard ratio for SARS-CoV-2 infection across several community factors among UUVS individuals in Cox proportional hazards model combining the three key community factors (S6 Table in S1 File). Persons living in counties with higher vaccine hesitancy (hazard ratio 1.25 [95% confidence interval 1.24–1.26] for medium and 1.26 [1.25–1.28] for high vaccine hesitant areas relative to patients living in low vaccine hesitant counties) and lower vaccination rates (1.13 [1.11–1.14] for low relative high county vaccination rates) had a higher risk of SARS-CoV-2 infection during the study period while limited association was observed based on rural residency. Univariate hazard ratios for all model covariates are available in S7 Table in S1 File.

### Multivariable-adjusted odds ratios for COVID-19 hospitalization and adverse events in persons based on vaccination status

**Fig 5:**
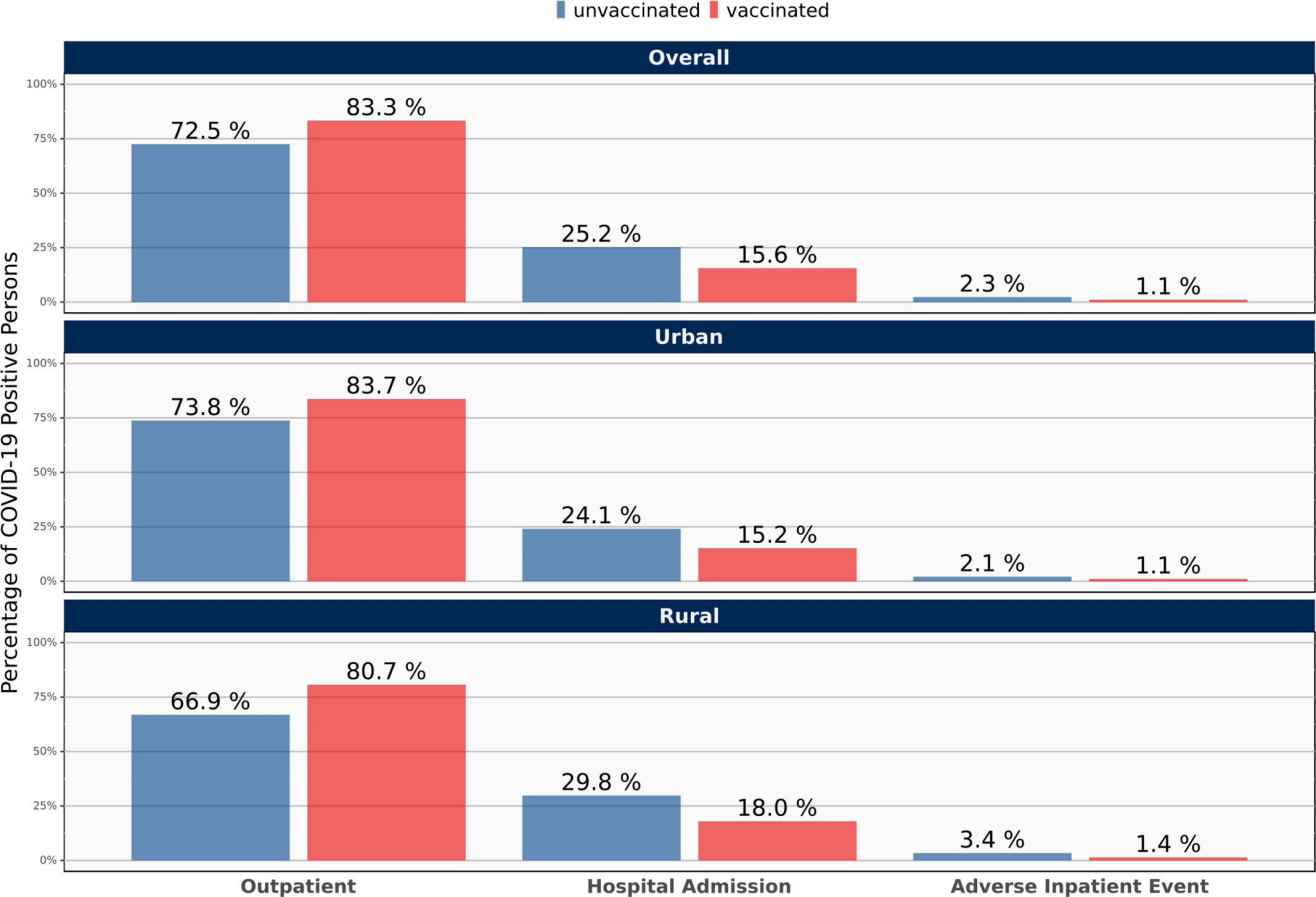
Differences in 30-day severity* based on vaccination status in persons infected with SARS-CoV-2, January 1, 2021 –December 20, 2021. *Severity categories following SARS-CoV-2 infection classified as outpatient-only visit (no hospitalization within 30 days of diagnosis), COVID-associated hospitalization (within 30 days of diagnosis), COVID-19-associated adverse event (mechanical ventilation, ECMO, or death or transfer to hospice within 30 days of diagnosis).

As shown in Fig 5, UUVS persons had higher rates of post-COVID hospitalization and adverse events (mechanical ventilation, ECMO, and death or transfer to hospice) within 30 days of infection. After adjusting for background risks and community factors, vaccinated patients had lower odds (adjusted odds ratio 0.58 [95% confidence interval 0.55–0.62] relative to UUVS patients) of hospitalization within 30 days of testing positive for SARS-CoV-2 and much lower odds (0.38 [0.30–0.46] relative to UUVS patients) of an adverse event within 30 days (S8 Table in S1 File). Increased adjusted odds ratios were observed for both hospitalization and adverse events based on key community factors. As shown in S8 Table in S1 File, demographic differences (notably older age, male gender, and race/ethnicity) and the presence of comorbid conditions were associated with increased odds of hospitalization and adverse events that were not observed for BTI.

## Discussion

Using a national database of 567,041 patients with evidence of having received 2 mRNA vaccine doses, we demonstrate that community factors play an important role in the risk of breakthrough SARS-CoV-2 infection amongst fully vaccinated individuals. Even after adjusting for patient-level factors (e.g., age, race, ethnicity, comorbid conditions, prior SARS-CoV-2 infections, and time of vaccination), the benefits of the COVID-19 vaccination remained independently associated with social determinants including rural dwelling, county vaccine hesitancy rates, and county vaccination rates. These factors should be considered when designing mitigation strategies to reduce the spread of COVID-19 in US communities averse to vaccines or those inaccessible to public health efforts.

The COVID-19 pandemic has revealed significant societal, gender and racial disparities in access to care, post-infectious complications, and economic hardships [36]. Rural and remote communities have been disproportionately impacted by the COVID-19 pandemic [37] in terms of increased morbidity and mortality, as well as negative impacts on employment rates, life satisfaction, mental health, and economic outlook [38]. Eighteen percent of the US population resides in the 84% of land area classified as rural by the US Federal Office of Research Health Policy [39]. Nonetheless, most research, public health resources [40], and healthcare expenditures [41, 42] have focused on urban centers, with far fewer studies considering the marginalizing effects of rurality on vulnerability to SARS-CoV-2 infection and downstream consequences.

Earlier studies have also demonstrated that COVID-19 mortality rates correlate with pre-existing social vulnerability and lower community resilience, defined as a community’s ability to respond and react to natural disasters, including the pandemic [43]. However, this is the first study to examine the risk of BTI related to these factors. Although we show that BTI rates were higher in rural than urban communities, there was an increased risk of breakthrough infection in both urban and rural communities with low vaccination rates. Reasons for this may include occupational and dwelling clusters in urban communities while vaccine hesitancy may be associated with lower compliance than non-vaccine risk mitigation strategies in rural communities [44]. One study examining disparate COVID-19 risk mitigation strategies between rural and urban dwellers demonstrated that rural community dwellers were less likely to social distance effectively, limit gatherings, avoid touching their face, and avoid contact with others outside their household; only not maintaining social distancing remained significant after adjusting for demographic factors [45].

As of April 3, 2022, 75.5% of US adults are fully vaccinated [46]. However, there is vast variability in the geographic distribution of vaccination rates between counties and communities, rendering certain regions much more vulnerable to COVID infection and local outbreaks [15, 46, 47]. Despite the proven benefit of vaccination against COVID-19 in reducing the probability of severe illness, hospitalization, and death, many Americans remain resistant or unwilling to consider vaccination. Hesitancy rates are higher amongst those residing in rural areas [15, 44], suggesting the need for policymakers to develop innovative strategies to address this disparity among rural dwellers [48]. Vaccination can directly reduce the risk of infection at an individual level, and may also provide community-level benefits [49].

Despite reduction in vaccine hesitancy over time [50], hesitancy still poses a challenge to providing broad coverage in some populations. Our analysis demonstrates that low vaccination rates in a community may increase the risk for SARS-CoV-2 infection among vaccinated as well as unvaccinated community members. Vaccine trials have demonstrated a vaccine efficacy of 95% in reducing breakthrough SARS-CoV-2 infection after a second dose of an mRNA vaccination, though the reported median follow-up was only 2 months [51]. However, the likelihood of acquiring COVID-19, even after completing two mRNA doses, is higher in communities with lower vaccination rates.

Earlier studies have suggested an increased overall COVID-19 risk in rural areas due to multiple factors including presence of fewer physicians, lack of mental health services, higher rates of disability, and higher proportions of uninsured persons, as well as having older populations with more comorbid conditions [52]. This study demonstrates a higher risk of breakthrough SARS-CoV-2 infection in these communities, but importantly, attributable complications and adverse outcomes are also expected to be more pronounced when COVID-19 occurs among rural dwellers. Although rural vaccinated patients from regions with high vaccine hesitancy remain at risk for BTI, they will likely still incur individual benefit from vaccination on account of the clearly reduced risk of adverse outcomes post-COVID diagnosis in vaccinated versus unvaccinated patients. Our findings show lower odds of breakthrough infection in those vaccinated with Moderna than Pfizer-BioNTech, which has been demonstrated in other large-scale studies [53], but that both mRNA vaccinations are equally protective against adverse outcomes. Rural dwellers were more likely to be vaccinated with Moderna (32% UAR and 31% NAR versus 37% urban), likely due to timing of vaccination when Moderna was more widely available. Despite this, rural dwellers were more likely to have breakthrough infections.

Importantly, this study highlights the fact that while vaccination against COVID-19 is the best strategy to mitigate poor outcomes, other important factors must be considered, particularly in rural regions with low vaccination rates. Not all community factors are likely to increase the risk of BTI, as observed in a sensitivity analysis using the disaster-preparedness composite Social Vulnerability Index score as a comparison test for falsifiability, so identifying potential populations for public health focus is an important step in reducing spread. Our data demonstrate that individuals receiving 2 doses of mRNA vaccination remain at higher risk for BTI in rural areas with relatively low vaccination rates and high vaccine hesitancy. Our findings suggest that prevention of BTI in rural areas of the US should not only develop and test strategies to improve vaccination rates but should also address methods to increase masking, proper hand hygiene, and social distancing. Increasing vaccination rates will require identifying barriers and facilitators to vaccination that may differ between urban and rural communities. Tailored education and outreach strategies from trusted thought leaders are critical to ensure maximal vaccination uptake. Further study and evaluation of community-based demonstration projects attempting to increase rural vaccination rates may inform the necessary conditions and approaches for success in overcoming vaccine hesitancy.

### Limitations

This study has notable limitations. N3C contains EHR data from multiple, diverse sites with differences in data reporting that may potentially result in misclassification of comorbid conditions and vaccination reporting based on degree of hospital interaction. We report similar comorbid burden as other national studies using more homogenous EHR-based data sources [54], but less is known about vaccination documentation using EHR data. While we anticipate non-differential misclassification, and likely underestimation of the impact of vaccination due to underreporting among communities at highest risk, we acknowledge that all comparisons are made to patients lacking documented vaccination status rather than documentation of unvaccinated status. We also note that the N3C demographic-matching process to include 2:1 negative controls: SARS-CoV-2 positive subjects preclude investigation of COVID-19 incidence [18], however we include data from individuals without reported vaccination to serve as a comparison for purposes of validating relative risk across these cohorts.

We selected a subset of participating organizations in N3C (described in S1 Methods in S1 File) to enrich for sites administering vaccinations or including data from state vaccine registries or health information exchanges. Despite this, we suspect that many data partners included in this study are not capturing all community-administered vaccinations, which likely results in an underestimation of both vaccination rates and asymptomatic infections. However, we believe this to be a strength of the study as vaccination is a long-term means to managing hospitalization and adverse events, which are captured in the EHR data submitted by participating organizations. Another limitation is that our analyses rely on public reporting (i.e., county vaccination rates), which has been inconsistent between local, state, and national tracking systems [55], and public surveys that have limitations given the sampling strategies [56]. We also note inconsistencies in data reporting, necessitating removal of patients with missing ZIP Codes, age, and gender. Finally, our study does not address BTI among persons receiving 3 mRNA vaccine doses due to small samples sizes likely resulting from the lag time in reporting to N3C by data partners.

## Conclusions

This retrospective cohort study utilizing real-world data documents differences in risk of breakthrough SARS-CoV-2 infection following vaccination based on community-derived risk factors. Understanding the role of community factors in preventing the spread of SARS-CoV-2 and reducing the risk of hospitalization and adverse events is crucial to controlling the pandemic as vaccination protection wanes. Our findings suggest that vaccinated persons dwelling in communities with certain characteristics such as high vaccine hesitancy are at a greater risk of BTI, despite being vaccinated, compared with persons in communities without those risk factors. Public health messaging should highlight this point and emphasize the high importance of precautionary measures (e.g., mask wearing, social distancing). In addition, thought leaders living in high-risk communities should be engaged in strategies to communicate the risk imposed by vaccine hesitancy and other associated characteristics, such as lower compliance with limiting gatherings and social distancing. Unfortunately, both vaccine hesitancy and lower compliance with such precautionary measures have become so politically charged in the US that public health messaging based solely on findings such as ours may not be as persuasive as, for example, appeals to protect the most vulnerable members of a community. To be most effective, public health interventions in some rural communities may benefit from input from multiple stakeholders, and community leaders working together with medical providers and public health officials to develop very targeted messages based on a community’s specific or unique characteristics.

## Supporting information

S1 File Supplementary online content—contains all the supporting tables and figures. (DOCX)

## Abbreviations

aHR: Adjusted Hazard Ratio
aOR: Adjusted Odds Ratio
BMT: Bone marrow transplant
BTI: Breakthrough infection
CDC: Center for Disease Control and Prevention
HIV: Human immunodeficiency virus
HR: Hazard ratio
ICD-10-CM: International Classification of Diseases, Tenth Revision, Clinical Modification
IQR: Interquartile range
IRB: Institutional review board
ISC: Immunosuppressed or immunocompromised state
MS: Multiple sclerosis
NAR: Nonurban-adjacent rural dwelling status
N3C: National COVID Cohort Collaborative
OMOP: Observational Medical Outcomes Partnership
RD: Autoimmune or inflammatory rheumatic diseases
SOT: Solid organ transplant
SVI: Social Vulnerability Index
UAR: Urban-adjacent rural dwelling status
UUVS: Unvaccinated or undocumented vaccination status
