# Supplementary material for: Community risks for SARS-CoV-2 infection among fully vaccinated US adults by rurality: A retrospective cohort study from the National COVID Cohort Collaborative
Source: PLoS One. 2023 Jan 5;18(1):e0279968. doi: 10.1371/journal.pone.0279968 (PMC9815650; doi:10.1371/journal.pone.0279968)
Supplement: S1 File — (DOCX) [file pone.0279968.s001.docx]

**Supplementary Online Content**

Anzalone AJ, Sun J, Vinson AJ, et al.; National COVID Cohort Collaborative (N3C) Consortium. *Community and Social Risks for SARS-CoV-2 Infection Among Fully Vaccinated US Adults: A Retrospective Cohort Study from the National COVID Cohort Collaborative*.

**S1 Fig.** Summary of Rural-Dwelling and Vaccination Distribution of 20 Data Partners Included in Study Sample

**S2 Fig.** Data Partner Data Censoring Date and Vaccine Censoring Date

**S3 Fig.** Analytical Sample Selection Flow Diagram

**S4 Fig.** Kaplan Meier Cumulative Incidence Curves for SARS-CoV-2 Breakthrough Infection for Patients in N3C Receiving 2 Doses of mRNA Vaccine Between January 1, 2021, and September 21, 2021, with Risk Tables

**S1 Table.** Baseline Characteristics of All Patients in N3C Without Prior SARS-CoV-2 Infection or a Documented Vaccination Administration Event Seen Between January 1, 2021, and September 21, 2021

**S2 Table.** Univariable Cox-Proportional Hazard Ratios for 180-Day Vaccine Breakthrough in Fully Vaccinated Adults, January 1, 2021 – December 20, 2021

**S3 Table.** Multivariable Cox-Proportional Hazard Ratios for 180-Day Vaccine Breakthrough in Fully Vaccinated Adults Separated by Key Exposure, January 1, 2021 – December 20, 2021

**S4 Table.** Multivariable Cox-Proportional Hazard Ratios for 180-Day Vaccine Breakthrough in Fully Vaccinated Adults in N3C Stratified by Rurality, January 1, 2021 – December 20, 2021

**S5 Table.** Sensitivity Analyses for Multivariable Cox-Proportional Hazard Ratios for 180-Day Vaccine Breakthrough in Fully Vaccinated Adults in N3C, January 2021 - December 2021

**S6 Table.** Multivariable Cox-Proportional Hazard Ratios for SARS-CoV-2 Infection in Unvaccinated or Undocumented Vaccination Status Adults, January 1, 2021 – December 20, 2021

**S7 Table.** Univariable Cox-Proportional Hazard Ratios for SARS-CoV-2 Infection in Unvaccinated or Undocumented Vaccination Status Adults, January 1, 2021 – December 20, 2021

**S8 Table.** Multivariable Odds Ratios for 30-Day Hospitalization or Adverse Event Following SARS-CoV-2 Infection in Persons Vaccinated and Unvaccinated or without Documented Vaccination Event, January 2021 - December 2021

**S1 Methods.** National COVID Cohort Collaborative (N3C) Methods

**S2 Methods.** List of Data Partners with Released or Pending Data in N3C

**Supplemental References**

This supplementary material has been provided by the authors to give readers additional information about their work.

**S1 Fig. Summary of Rural-Dwelling and Vaccination Distribution of 20 Data Partners Included in Study Sample**

**
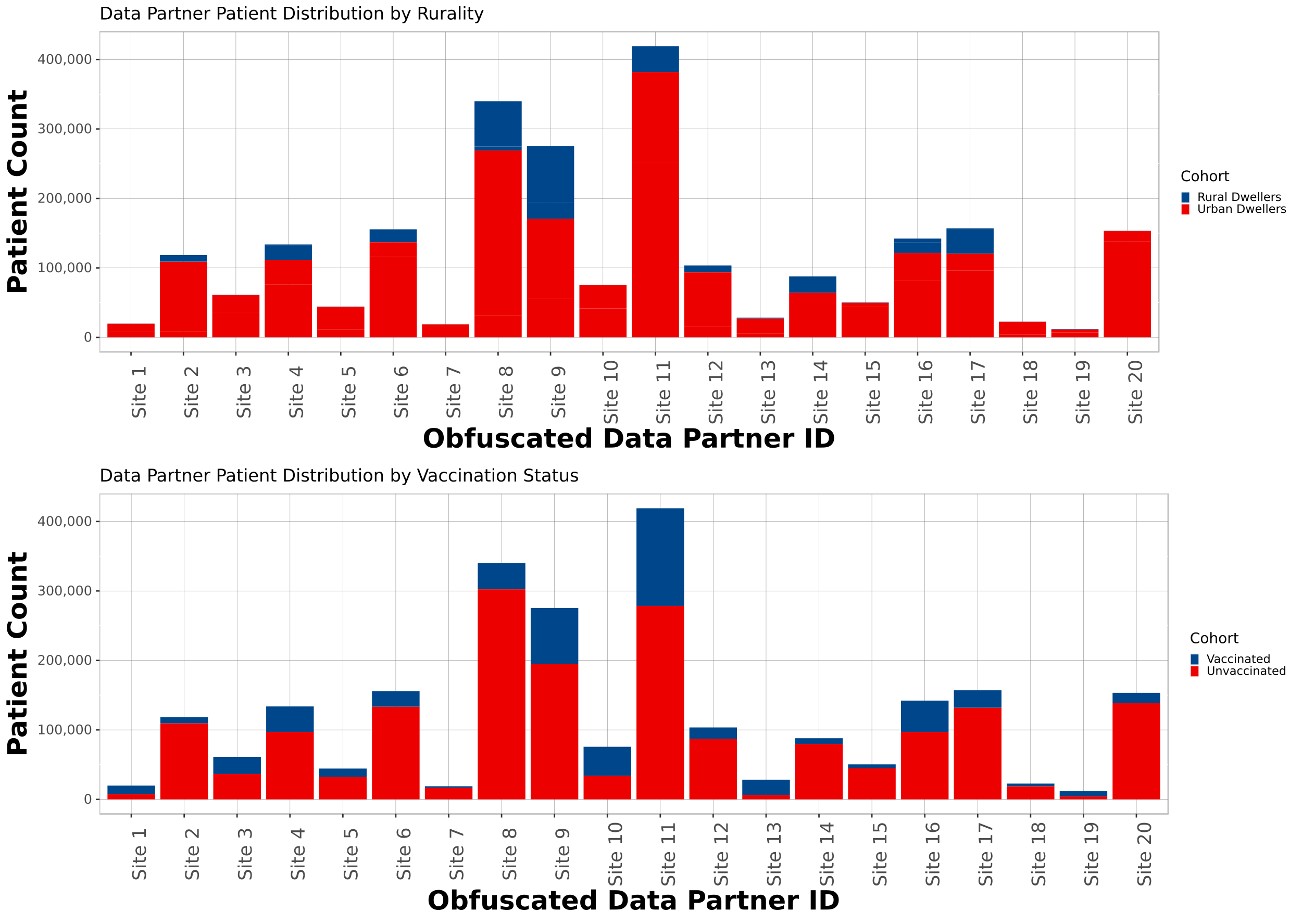
**

**S2 Fig. Data Partner Data Censoring Date and Vaccine Censoring Date**

**
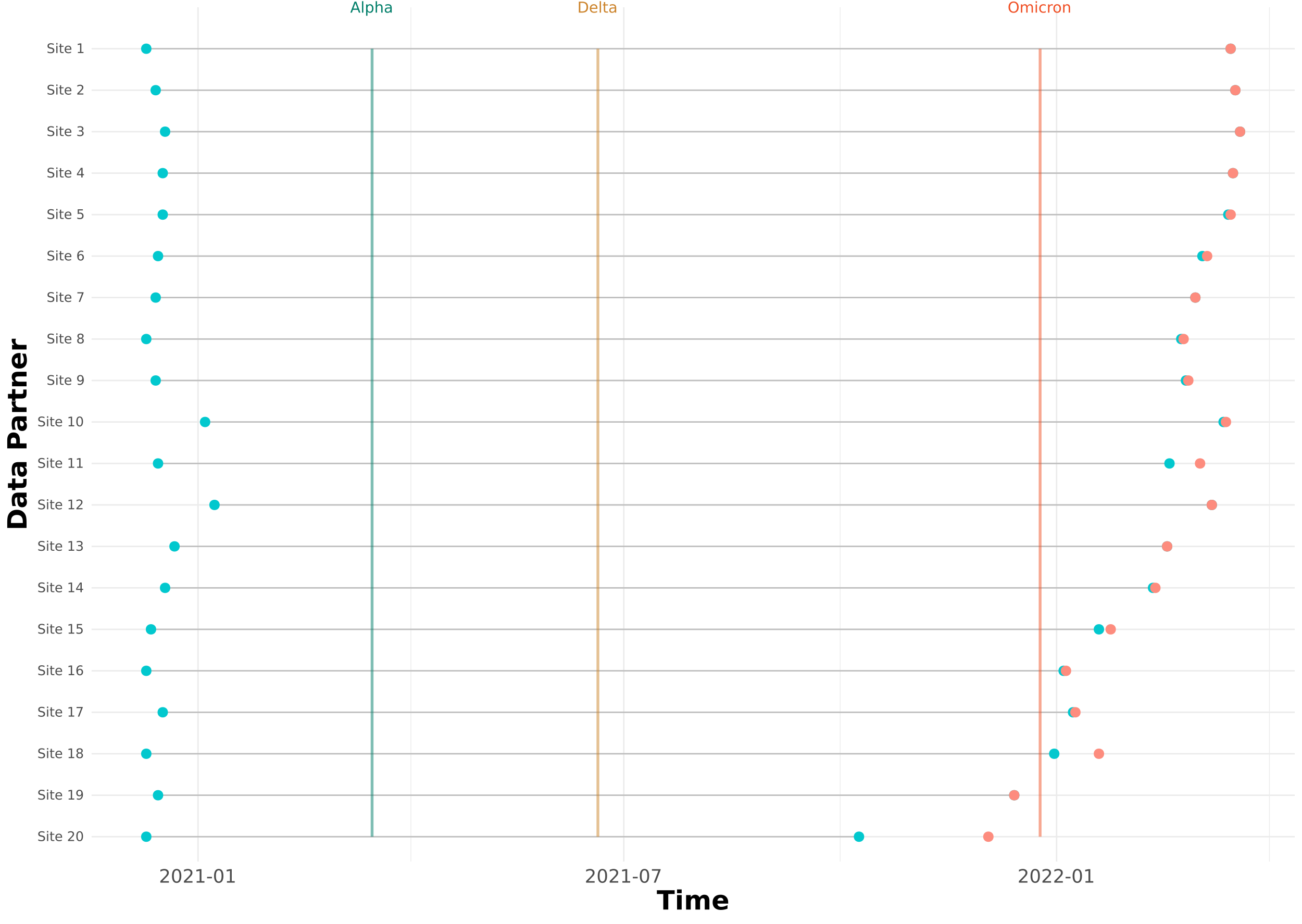
**

*****Data latency varies by site. eFigure 2 shows vaccination range and individual site censor date, which represents the final visit history deposited at release 71.

**S3 Fig.** Analytical Sample Selection Flow Diagram

**
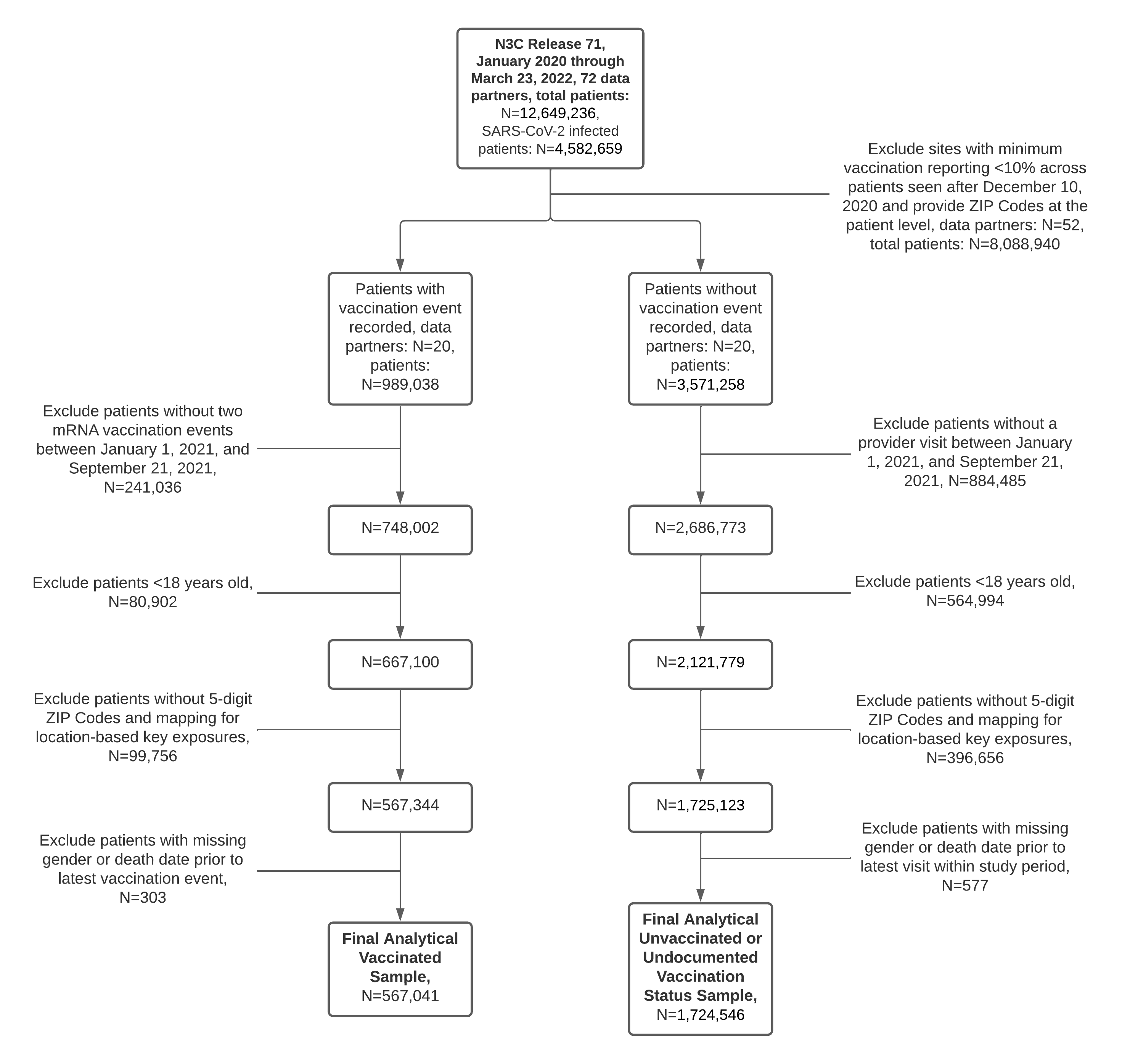
**

**S4 Fig. Kaplan Meier Cumulative Incidence Curves for SARS-CoV-2 Breakthrough Infection for Patients in N3C Receiving 2 Doses of mRNA Vaccine Between January 1, 2021, and September 21, 2021, with Risk Tables**

**A. Stratified by Rural-Urban Dwelling Status**


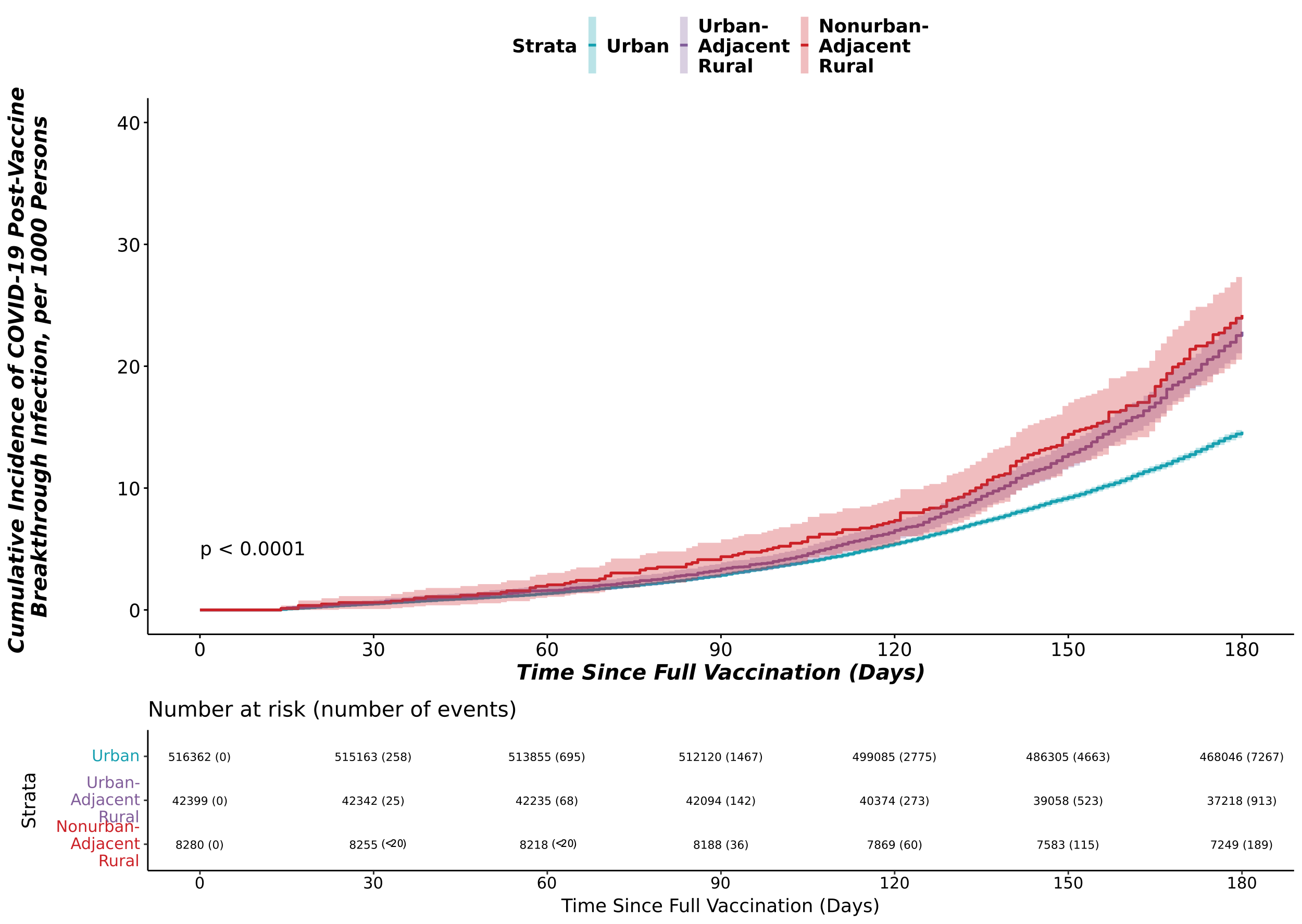


**B. Stratified by Community Vaccine Hesitancy Levels**


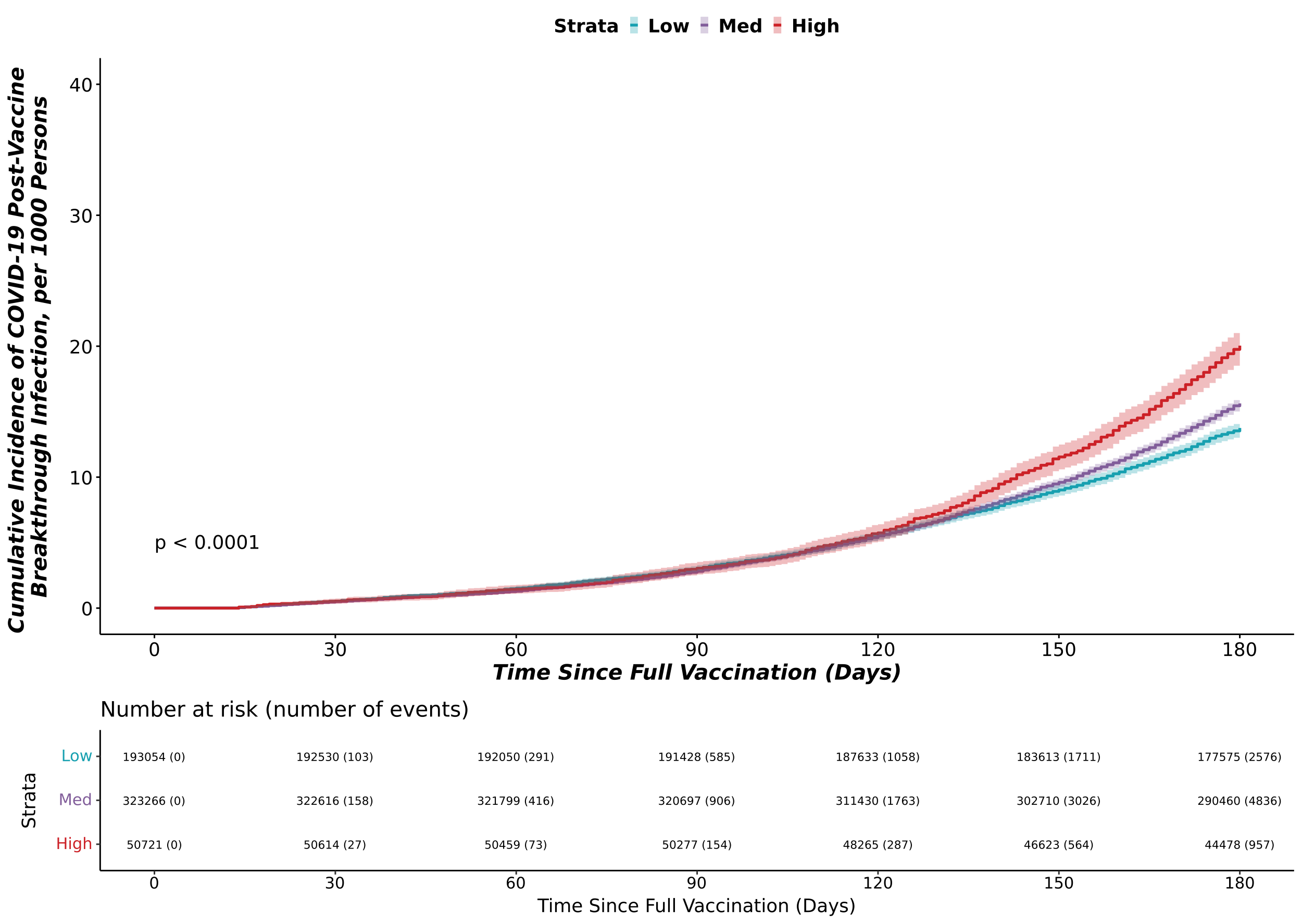


**C. Stratified by County Vaccination Rates through September 21, 2021**


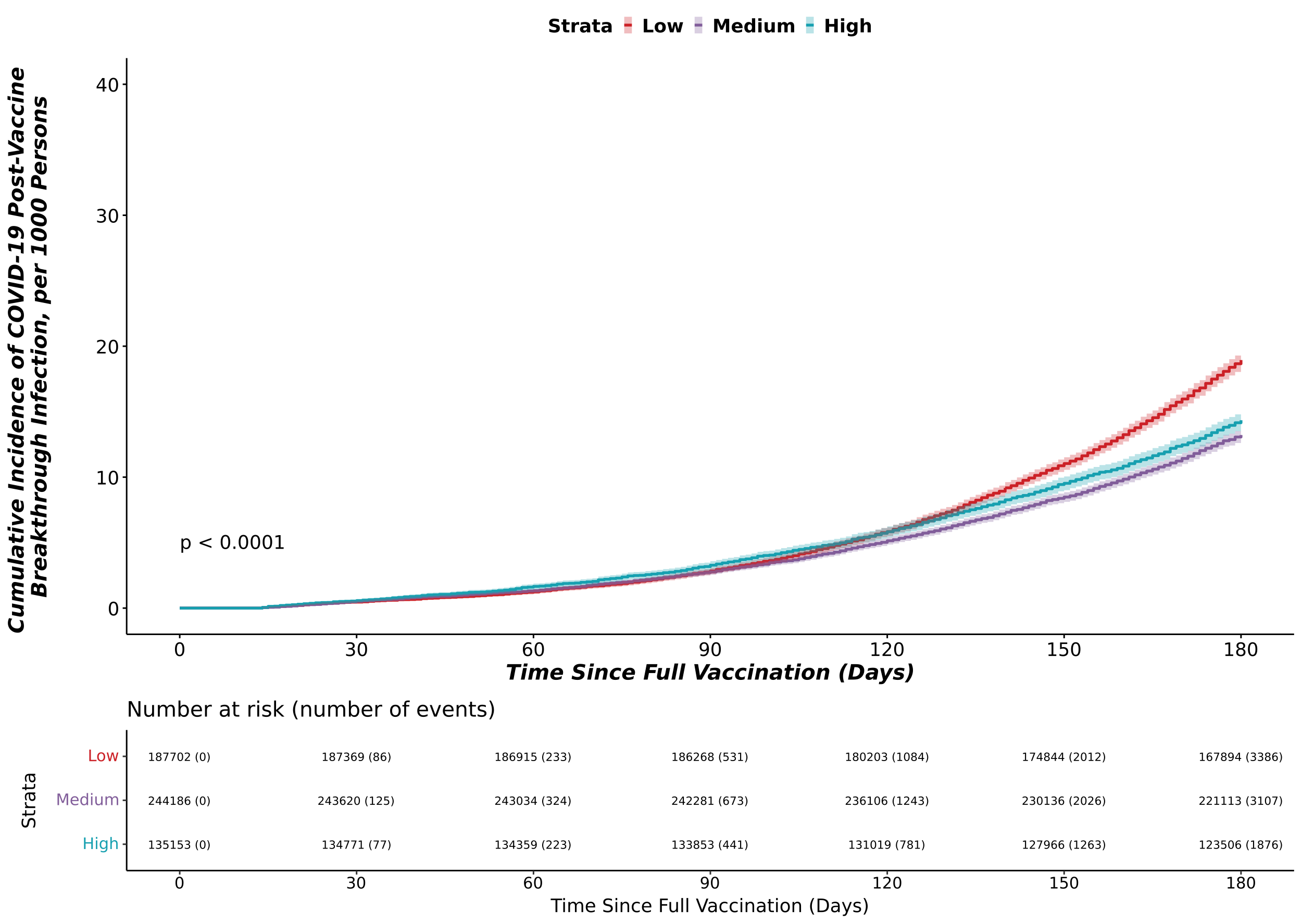


**S1 Table. Baseline Characteristics of All Patients in N3C Without Prior SARS-CoV-2 Infection or a Documented Vaccination Administration Event Seen Between January 1, 2021, and September 21, 2021**

| **Characteristic** | **Overall,**  N = 1,724,546^1^ |  | **Rural Dwelling Status^2^** | | | |
| --- | --- | --- | --- | --- | --- | --- |
|  |  |  | **Urban,**  N=1,435,374^1^ | **Urban-Adjacent Rural,**  N=234,891^1^ | **Nonurban-Adjacent Rural,**  N=54,281^1^ | ***P* value**^2^ |
| SARS-CoV-2 Infection (PCR, AG, or Condition Diagnosis) During Study Period | 343,348 (20%) |  | 279,545 (19%) | 51,873 (22%) | 11,930 (22%) | <0.001 |
| SARS-CoV-2 Infection (PCR, AG, or Condition Diagnosis) Prior to Study Period | 162,330 (9.4%) |  | 137,565 (9.6%) | 20,393 (8.7%) | 4,372 (8.1%) | <0.001 |
| Age, Median (IQR) | 46 (32, 61) |  | 45 (31, 61) | 50 (34, 64) | 53 (36, 66) | <0.001 |
| Age Category | |  |  | | | <0.001 |
| <30 | 362,301 (21%) |  | 311,195 (22%) | 42,383 (18%) | 8,723 (16%) |  |
| 30-49 | 591,240 (34%) |  | 502,080 (35%) | 73,519 (31%) | 15,641 (29%) |  |
| 50-64 | 427,101 (25%) |  | 349,658 (24%) | 62,267 (27%) | 15,176 (28%) |  |
| 65-75 | 219,627 (13%) |  | 173,036 (12%) | 37,009 (16%) | 9,582 (18%) |  |
| >75 | 124,277 (7.2%) |  | 99,405 (6.9%) | 19,713 (8.4%) | 5,159 (9.5%) |  |
| Gender | |  |  | | | <0.001 |
| Female | 991,990 (58%) |  | 832,184 (58%) | 130,243 (55%) | 29,563 (54%) |  |
| Male | 732,556 (42%) |  | 603,190 (42%) | 104,648 (45%) | 24,718 (46%) |  |
| Race/Ethnicity | |  |  | | | <0.001 |
| Non-Hispanic/Latinx White | 1,072,699 (62%) |  | 848,802 (59%) | 179,294 (76%) | 44,603 (82%) |  |
| Non-Hispanic/Latinx Black | 218,481 (13%) |  | 194,027 (14%) | 20,235 (8.6%) | 4,219 (7.8%) |  |
| Hispanic/Latinx | 191,495 (11%) |  | 171,145 (12%) | 17,635 (7.5%) | 2,715 (5.0%) |  |
| Asian American/Pacific Islander | 49,941 (2.9%) |  | 47,848 (3.3%) | 1,909 (0.8%) | 184 (0.3%) |  |
| Other | 135,445 (7.9%) |  | 122,961 (8.6%) | 10,773 (4.6%) | 1,711 (3.2%) |  |
| Missing/Unknown | 56,485 (3.3%) |  | 50,591 (3.5%) | 5,045 (2.1%) | 849 (1.6%) |  |
| Vaccine Hesitancy, Median (IQR) | 0.09 (0.06, 0.14) |  | 0.09 (0.06, 0.12) | 0.16 (0.12, 0.20) | 0.18 (0.13, 0.22) | <0.001 |
| Vaccine Hesitancy Category^3^ | |  |  | | | <0.001 |
| Low (≤ 5% hesitant) | 433,113 (25%) |  | 419,284 (29%) | 11,863 (5.1%) | 1,966 (3.6%) |  |
| Medium (6-15% hesitant) | 941,440 (55%) |  | 825,864 (58%) | 97,671 (42%) | 17,905 (33%) |  |
| High (>15% hesitant) | 349,993 (20%) |  | 190,226 (13%) | 125,357 (53%) | 34,410 (63%) |  |
| County Vaccination Rate^3^ | 71 (63, 75) |  | 74 (67, 76) | 60 (54, 67) | 58 (52, 64) | <0.001 |
| County Vaccination Rate Category^3^ | |  |  | | | <0.001 |
| Low (<70%) | 827,365 (48%) |  | 570,385 (40%) | 207,186 (88%) | 49,794 (92%) |  |
| Medium (70-77%) | 660,647 (38%) |  | 642,110 (45%) | 16,412 (7.0%) | 2,125 (3.9%) |  |
| High (>77%) | 236,534 (14%) |  | 222,879 (16%) | 11,293 (4.8%) | 2,362 (4.4%) |  |
| U.S. Census Region | |  |  | | | <0.001 |
| Northeast | 164,740 (9.6%) |  | 163,581 (11%) | 750 (0.3%) | 409 (0.8%) |  |
| Midwest | 592,824 (34%) |  | 464,672 (32%) | 102,089 (43%) | 26,063 (48%) |  |
| South | 515,456 (30%) |  | 402,512 (28%) | 93,529 (40%) | 19,415 (36%) |  |
| West | 451,526 (26%) |  | 404,609 (28%) | 38,523 (16%) | 8,394 (15%) |  |
| Charlson Comorbidity Index, Median (IQR) | 0.00 (0.00, 2.00) |  | 0.00 (0.00, 2.00) | 1.00 (0.00, 2.00) | 1.00 (0.00, 3.00) | <0.001 |
| Number of Comorbid Conditions | |  |  | | | <0.001 |
| 0 Comorbid Conditions | 918,015 (53%) |  | 782,618 (55%) | 111,051 (47%) | 24,346 (45%) |  |
| 1 Comorbid Conditions | 401,181 (23%) |  | 333,871 (23%) | 54,199 (23%) | 13,111 (24%) |  |
| 2 Comorbid Conditions | 185,606 (11%) |  | 149,223 (10%) | 29,230 (12%) | 7,153 (13%) |  |
| >=3 Comorbid Conditions | 219,744 (13%) |  | 169,662 (12%) | 40,411 (17%) | 9,671 (18%) |  |
| Comorbid Conditions^3^ | |  |  | | | |
| Heart Disease | 164,310 (9.5%) |  | 125,255 (8.7%) | 31,290 (13%) | 7,765 (14%) | <0.001 |
| Peripheral Vascular Disease | 126,166 (7.3%) |  | 99,088 (6.9%) | 21,748 (9.3%) | 5,330 (9.8%) | <0.001 |
| Peptic Ulcer Disease | 31,737 (1.8%) |  | 25,270 (1.8%) | 5,196 (2.2%) | 1,271 (2.3%) | <0.001 |
| Mild or Severe Liver Disease | 132,049 (7.7%) |  | 106,972 (7.5%) | 20,471 (8.7%) | 4,606 (8.5%) | <0.001 |
| Diabetes Mellitus | 305,292 (18%) |  | 242,827 (17%) | 50,504 (22%) | 11,961 (22%) | <0.001 |
| Hemiplegia or Paraplegia | 23,613 (1.4%) |  | 18,134 (1.3%) | 4,442 (1.9%) | 1,037 (1.9%) | <0.001 |
| Stroke | 122,386 (7.1%) |  | 95,422 (6.6%) | 21,835 (9.3%) | 5,129 (9.4%) | <0.001 |
| Renal Disease | 132,296 (7.7%) |  | 102,158 (7.1%) | 24,200 (10%) | 5,938 (11%) | <0.001 |
| Any Cancer (Except Skin) | 167,667 (9.7%) |  | 132,070 (9.2%) | 28,493 (12%) | 7,104 (13%) | <0.001 |
| Documented Obesity Before Vaccination | 608,533 (35%) |  | 488,674 (34%) | 96,609 (41%) | 23,250 (43%) | <0.001 |
| ISC Status (RD, SOT, MS, HIV, BMT) | 162,840 (9.4%) |  | 132,285 (9.2%) | 24,518 (10%) | 6,037 (11%) | <0.001 |
| 1. Statistics presented: n (%), Median (Interquartile Range [IQR])  2. Wilcoxon Rank Sum Test; Pearson’s Chi Squared Test  3. Definitions and logic used for rural categorization, vaccine hesitancy, county vaccination, and comorbid conditions provided in **eMethods 1** | | | | | | |

**S2 Table. Univariable Cox-Proportional Hazard Ratios for 180-Day Vaccine Breakthrough in Fully Vaccinated Adults, January 1, 2021 – December 20, 2021**

| **Covariate** | **Hazard Ratio**  **(95% Confidence Interval)** | **p value** |
| --- | --- | --- |
| Rurality^1^ |  |  |
| Urban | Reference |  |
| Urban-Adjacent Rural | 1.56 (1.45, 1.67) | <0.001 |
| Nonurban-Adjacent Rural | 1.65 (1.43, 1.91) | <0.001 |
| Vaccine Hesitancy Category^1^ |  |  |
| Low (≤ 5% hesitant) | Reference |  |
| Medium (6-15% hesitant) | 1.13 (1.08, 1.19) | <0.001 |
| High (>15% hesitant) | 1.45 (1.35, 1.56) | <0.001 |
| County Vaccination Rates1 |  |  |
| Low (<70%) | 1.31 (1.24, 1.39) | <0.001 |
| Medium (70-77%) | 0.92 (0.87, 0.97) | 0.004 |
| High (>77%) | Reference |  |
| Age Group |  |  |
| <30 | 0.82 (0.77, 0.88) | <0.001 |
| 30-49 | Reference |  |
| 50-64 | 0.90 (0.85, 0.95) | <0.001 |
| 65-75 | 0.70 (0.66, 0.75) | <0.001 |
| >75 | 0.65 (0.60, 0.71) | <0.001 |
| Gender |  |  |
| Female | Reference |  |
| Male | 1.08 (1.03, 1.12) | <0.001 |
| Race/Ethnicity |  |  |
| Non-Hispanic/Latinx White | Reference |  |
| Non-Hispanic/Latinx Black | 0.83 (0.77, 0.90) | <0.001 |
| Hispanic/Latinx | 0.87 (0.81, 0.93) | <0.001 |
| Asian American/Pacific Islander | 0.82 (0.74, 0.91) | <0.001 |
| Other | 0.90 (0.83, 0.98) | 0.019 |
| Missing/Unknown | 0.62 (0.53, 0.73) | <0.001 |
| Comorbid Conditions^1^ |  |  |
| Heart Disease | 0.99 (0.92, 1.07) | 0.8 |
| Peripheral Vascular Disease | 0.98 (0.92, 1.06) | 0.7 |
| Peptic Ulcer Disease | 1.00 (0.85, 1.16) | >0.9 |
| Mild or Severe Liver Disease | 0.95 (0.87, 1.03) | 0.2 |
| Diabetes Mellitus | 0.95 (0.90, 1.01) | 0.086 |
| Hemiplegia or Paraplegia | 0.92 (0.74, 1.15) | 0.5 |
| Stroke | 0.84 (0.77, 0.91) | <0.001 |
| Renal Disease | 1.11 (1.03, 1.19) | 0.004 |
| Any Cancer (Except Skin) | 0.85 (0.80, 0.92) | <0.001 |
| Documented Obesity | 1.16 (1.11, 1.21) | <0.001 |
| ISC (RD, SOT, MS, HIV, BMT) | 1.31 (1.23, 1.39) | <0.001 |
| Vaccine Manufacturer |  |  |
| Pfizer BioNTech | Reference |  |
| Moderna NIAID | 0.74 (0.70, 0.78) | <0.001 |
| Time Period of Second Vaccination Administration |  |  |
| Pre-Delta (< June 20, 2021) | Reference |  |
| Post-Delta (>= June 20, 2021) | 2.32 (2.17, 2.48) | <0.001 |
| Pre-Vaccine SARS-CoV-2 Infection History |  |  |
| No Reported Hx of SARS-CoV-2 | Reference |  |
| Reported Hx of SARS-CoV-2 | 0.22 (0.20, 0.24) | <0.001 |
| Region |  |  |
| Northeast | Reference |  |
| Midwest | 1.64 (1.52, 1.77) | <0.001 |
| South | 1.19 (1.09, 1.30) | <0.001 |
| West | 1.71 (1.59, 1.83) | <0.001 |
| 1. Definitions and logic used for rural categorization, vaccine hesitancy categories, Social Deprivation Index, county vaccination rates, and comorbid conditions provided in eMethods S-1. | | |

**S3 Table. Multivariable Cox-Proportional Hazard Ratios for 180-Day Vaccine Breakthrough in Fully Vaccinated Adults Separated by Key Exposure, January 1, 2021 – December 20, 2021**

| **Covariate** | **Model 1: Rurality**  **Hazard Ratio (95% CI)^1^** | **Model 2: Vaccine Hesitancy**  **Hazard Ratio (95% CI)^1^** | **Model 3: County Vaccination Rate**  **Hazard Ratio (95% CI)^1^** |
| --- | --- | --- | --- |
| Primary  Exposure  Group^2^ | Urban  Reference | Low (≤5%)  Reference | Low (<70%)  1.34 (1.27, 1.43) |
|  | Urban-Adjacent Rural  1.53 (1.42, 1.64) | Medium (6-15%)  1.07 (1.02, 1.12) | Medium (70-77%)  1.00 (0.95, 1.07) |
|  | Nonurban-Adjacent Rural  1.65 (1.42, 1.91) | High (>15%)  1.33 (1.23, 1.43) | High (>77%)  Reference |
| Age Group | | | |
| <30 | 0.86 (0.81, 0.93) | 0.86 (0.80, 0.92) | 0.85 (0.80, 0.91) |
| 30-49 | Reference | Reference | Reference |
| 50-64 | 0.87 (0.82, 0.92) | 0.87 (0.82, 0.92) | 0.87 (0.82, 0.92) |
| 65-75 | 0.65 (0.61, 0.70) | 0.66 (0.61, 0.70) | 0.66 (0.61, 0.71) |
| >75 | 0.58 (0.53, 0.64) | 0.59 (0.54, 0.65) | 0.60 (0.55, 0.66) |
| Gender | | | |
| Female | Reference | Reference | Reference |
| Male | 1.09 (1.05, 1.14) | 1.10 (1.05, 1.15) | 1.10 (1.05, 1.15) |
| Race/Ethnicity | | | |
| Non-Hispanic/Latinx White | Reference | Reference | Reference |
| Non-Hispanic/Latinx Black | 0.98 (0.90, 1.06) | 0.94 (0.86, 1.02) | 0.97 (0.90, 1.06) |
| Hispanic/Latinx | 1.02 (0.95, 1.09) | 1.00 (0.94, 1.08) | 1.02 (0.95, 1.09) |
| Asian American/Pacific Islander | 0.90 (0.81, 1.00) | 0.89 (0.80, 0.99) | 0.89 (0.80, 0.99) |
| Other | 1.04 (0.96, 1.14) | 1.03 (0.94, 1.12) | 1.03 (0.95, 1.13) |
| Missing/Unknown | 0.75 (0.64, 0.89) | 0.75 (0.64, 0.88) | 0.75 (0.64, 0.89) |
| Comorbid Conditions**^2^** | | | |
| Heart Disease | 1.02 (0.94, 1.11) | 1.01 (0.93, 1.10) | 1.01 (0.93, 1.10) |
| Peripheral Vascular Disease | 1.07 (0.99, 1.16) | 1.07 (0.99, 1.16) | 1.06 (0.98, 1.15) |
| Peptic Ulcer Disease | 0.98 (0.84, 1.15) | 0.98 (0.83, 1.14) | 0.97 (0.83, 1.14) |
| Mild or Severe Liver Disease | 0.90 (0.83, 0.98) | 0.89 (0.82, 0.97) | 0.89 (0.82, 0.96) |
| Diabetes Mellitus | 0.98 (0.92, 1.04) | 0.97 (0.91, 1.03) | 0.97 (0.91, 1.03) |
| Hemiplegia or Paraplegia | 0.95 (0.75, 1.19) | 0.94 (0.75, 1.18) | 0.94 (0.75, 1.18) |
| Stroke | 0.86 (0.79, 0.95) | 0.86 (0.78, 0.94) | 0.86 (0.78, 0.94) |
| Renal Disease | 1.18 (1.08, 1.28) | 1.18 (1.09, 1.28) | 1.18 (1.09, 1.29) |
| Any Cancer (Except Skin) | 0.87 (0.81, 0.94) | 0.87 (0.81, 0.94) | 0.87 (0.81, 0.94) |
| Documented Obesity | 1.14 (1.09, 1.20) | 1.14 (1.08, 1.19) | 1.14 (1.08, 1.19) |
| ISC (RD, SOT, MS, HIV, BMT) | 1.32 (1.24, 1.41) | 1.32 (1.24, 1.41) | 1.32 (1.24, 1.41) |
| Vaccine Manufacturer | | | |
| Pfizer BioNTech | Reference | Reference | Reference |
| Moderna NIAID | 0.78 (0.74, 0.83) | 0.80 (0.76, 0.85) | 0.79 (0.75, 0.83) |
| Time Period of Second Vaccination Administration | | | |
| Pre-Delta (< June 20, 2021) | Reference | Reference | Reference |
| Post-Delta (>= June 20, 2021) | 1.19 (1.10, 1.29) | 1.20 (1.11, 1.30) | 1.21 (1.11, 1.31) |
| Pre-Vaccine SARS-CoV-2 Infection History | | | |
| No Reported Hx of SARS-CoV-2 | Reference | Reference | Reference |
| Reported Hx of SARS-CoV-2 | 0.25 (0.22, 0.29) | 0.25 (0.22, 0.29) | 0.25 (0.22, 0.29) |
| US Census Region | | | |
| Northeast | Reference | Reference | Reference |
| Midwest | 1.22 (1.12, 1.32) | 1.27 (1.17, 1.38) | 1.15 (1.06, 1.25) |
| South | 0.97 (0.89, 1.06) | 0.98 (0.89, 1.07) | 0.88 (0.80, 0.96) |
| West | 1.39 (1.29, 1.50) | 1.39 (1.29, 1.50) | 1.32 (1.22, 1.42) |
| 1. Models separated by primary exposure to assess independent adjusted risk of rurality, vaccine hesitancy, community vaccination rate, community period incidence, and Social Vulnerability Index on post-vaccination SARS-CoV-2 infection.  2. Definitions and logic used for rural categorization, vaccine hesitancy categories, Social Deprivation Index, county vaccination rates, and comorbid conditions provided in eMethods S-1. | | | |

**S4 Table. Multivariable Cox-Proportional Hazard Ratios for 180-Day Vaccine Breakthrough in Fully Vaccinated Adults in N3C Stratified by Rurality, January 1, 2021 – December 20, 2021**

| **Covariate** | **Model 1: Urban Dwellers**  **Hazard Ratio**  **(95% Confidence Interval)** | **Model 2: Rural Dwellers**  **Hazard Ratio**  **(95% Confidence Interval)** |
| --- | --- | --- |
| Vaccine Hesitancy Category^1^ | | |
| Low (≤ 5% hesitant) | Reference | Reference |
| Medium (6-15% hesitant) | 0.97 (0.93, 1.03) | 1.22 (0.94, 1.60) |
| High (>15% hesitant) | 0.92 (0.83, 1.03) | 1.25 (0.94, 1.65) |
| County Vaccination Rates^1^ | | |
| Low (<70% vaccinated) | 1.30 (1.21, 1.39) | 1.17 (0.91, 1.49) |
| Medium (70-77% vaccinated) | 0.98 (0.92, 1.04) | 1.18 (0.90, 1.54) |
| High (>77% vaccinated) | Reference | Reference |
| Age Group | | |
| <30 | 0.88 (0.82, 0.95) | 0.71 (0.57, 0.88) |
| 30-49 | Reference | Reference |
| 50-64 | 0.87 (0.82, 0.92) | 0.86 (0.74, 1.01) |
| 65-75 | 0.65 (0.60, 0.70) | 0.72 (0.59, 0.87) |
| >75 | 0.56 (0.51, 0.62) | 0.71 (0.57, 0.88) |
| Gender | | |
| Female | Reference | Reference |
| Male | 1.09 (1.04, 1.14) | 1.15 (1.02, 1.30) |
| Race/Ethnicity | | |
| Non-Hispanic/Latinx White | Reference | Reference |
| Non-Hispanic/Latinx Black | 1.04 (0.95, 1.13) | 1.03 (0.69, 1.53) |
| Hispanic/Latinx | 1.02 (0.95, 1.10) | 1.15 (0.92, 1.45) |
| Asian American/Pacific Islander | 0.90 (0.81, 1.01) | 1.19 (0.72, 1.96) |
| Other | 1.06 (0.97, 1.16) | 0.87 (0.58, 1.31) |
| Missing/Unknown | 0.78 (0.66, 0.92) | 0.54 (0.24, 1.21) |
| Comorbid Conditions | | |
| Heart Disease | 1.06 (0.97, 1.16) | 0.83 (0.66, 1.04) |
| Peripheral Vascular Disease | 1.11 (1.02, 1.20) | 0.76 (0.60, 0.96) |
| Peptic Ulcer Disease | 0.89 (0.74, 1.06) | 1.58 (1.12, 2.24) |
| Mild or Severe Liver Disease | 0.91 (0.84, 1.00) | 0.76 (0.58, 0.99) |
| Diabetes Mellitus | 0.97 (0.90, 1.03) | 1.04 (0.89, 1.22) |
| Hemiplegia or Paraplegia | 0.95 (0.75, 1.21) | 0.95 (0.48, 1.84) |
| Stroke | 0.86 (0.78, 0.95) | 0.88 (0.69, 1.13) |
| Renal Disease | 1.19 (1.08, 1.30) | 1.05 (0.85, 1.29) |
| Malignant Neoplasm | 0.90 (0.83, 0.97) | 0.73 (0.59, 0.90) |
| Documented Obesity | 1.12 (1.07, 1.18) | 1.16 (1.02, 1.31) |
| ISC (RD, SOT, MS, HIV, BMT) | 1.32 (1.23, 1.42) | 1.31 (1.10, 1.56) |
| Vaccine Manufacturer | | |
| Pfizer BioNTech | Reference | Reference |
| Moderna NIAID | 0.81 (0.76, 0.86) | 0.68 (0.58, 0.80) |
| Time Period of Event or Censor Date | | |
| Pre-Delta (< June 20, 2021) | Reference | Reference |
| Post-Delta (>= June 20, 2021) | 1.20 (1.10, 1.30) | 1.01 (0.75, 1.36) |
| Pre-Vaccine SARS-CoV-2 Infection | | |
| No Documented Hx of SARS-CoV-2 Infection | Reference | Reference |
| Documented Hx of SARS-CoV-2 Infection | 0.25 (0.22, 0.29) | 0.22 (0.15, 0.34) |
| US Census Region | | |
| Northeast | Reference | Reference |
| Midwest | 1.03 (0.94, 1.12) | 2.71 (0.67, 10.9) |
| South | 0.93 (0.85, 1.03) | 1.08 (0.26, 4.41) |
| West | 1.35 (1.25, 1.46) | 2.06 (0.51, 8.32) |
| 1. Definitions and logic used for rural categorization, vaccine hesitancy categories, Social Deprivation Index, county vaccination rates, and comorbid conditions provided in eMethods S-1. | | |

**S5 Table. Sensitivity Analyses for Multivariable Cox-Proportional Hazard Ratios for 180-Day Vaccine Breakthrough in Fully Vaccinated Adults in N3C, January 2021 - December 2021**

| **Covariate** | **Sensitivity Analysis 1: Altered Hesitancy Categories**  **Hazard Ratio (95% CI)** | **Sensitivity Analysis 2:**  **Remove Patients with Prior SARS-CoV-2 Infection**  **Hazard Ratio (95% CI)** | **Sensitivity Analysis 3: Data Partner as Random Effect in Model**  **Hazard Ratio (95% CI)** | **Sensitivity Analysis 4: Rurality * Vaccine Hesitancy Interaction Terms**  **Hazard Ratio (95% CI)** | **Sensitivity Analysis 5: Inclusion of Social Vulnerability Index**  **Hazard Ratio (95% CI)** |
| --- | --- | --- | --- | --- | --- |
| Rurality^1^ |  |  |  |  |  |
| Urban | Reference | Reference | Reference | Reference | N/A |
| Urban-Adjacent Rural | 1.39 (1.29, 1.50) | 1.41 (1.31, 1.53) | 1.23 (1.15, 1.31) | 1.00 (0.79, 1.28) | N/A |
| Nonurban-Adjacent Rural | 1.50 (1.29, 1.74) | 1.48 (1.26, 1.72) | 1.24 (1.08, 1.39) | 0.35 (0.11, 1.09) | N/A |
| Vaccine Hesitancy Category^1^ |  |  |  |  |  |
| Low (≤ 5% hesitant) | N/A | Reference | Reference | Reference | N/A |
| Medium (6-15% hesitant) | N/A | 1.00 (0.95, 1.05) | 1.11 (1.05, 1.17) | 0.98 (0.93, 1.03) | N/A |
| High (>15% hesitant) | N/A | 1.00 (0.91, 1.09) | 1.17 (1.07, 1.27) | 0.95 (0.86, 1.05) | N/A |
| Altered Vaccine Hesitancy Category^1^ |  |  |  |  |  |
| Low (<10% hesitant) | Reference | N/A | N/A | N/A | N/A |
| Medium (10-15% hesitant) | 1.06 (1.01, 1.13) | N/A | N/A | N/A | N/A |
| High (>15% hesitant) | 1.03 (0.94, 1.11) | N/A | N/A | N/A | N/A |
| County Vaccination Rates^1^ |  |  |  |  |  |
| Low (<70%) | 1.25 (1.17, 1.33) | 1.27 (1.19, 1.35) | 1.40 (1.33, 1.48) | 1.28 (1.20, 1.36) | N/A |
| Medium (70-77%) | 1.00 (0.95, 1.06) | 1.00 (0.94, 1.06) | 1.17 (1.10, 1.24) | 1.00 (0.95, 1.06) | N/A |
| High (>77%) | Reference | Reference | Reference | Reference | N/A |
| Social Vulnerability Index^1^ |  |  |  |  |  |
| Low (<0.334) | N/A | N/A | N/A | N/A | Reference |
| Medium (0.334-0.666) | N/A | N/A | N/A | N/A | 1.02 (0.98, 1.08) |
| High (>0.666) | N/A | N/A | N/A | N/A | 1.08 (1.00, 1.18) |
| Rurality*Vaccine Hesitancy Categories |  |  |  |  |  |
| Urban-Adjacent Rural * Medium Vaccine Hesitancy | N/A | N/A | N/A | 1.52 (1.17, 1.98) | N/A |
| Nonurban-Adjacent Rural * Medium Vaccine Hesitancy | N/A | N/A | N/A | 3.72 (1.17, 11.9) | N/A |
| Urban-Adjacent Rural * High Vaccine Hesitancy | N/A | N/A | N/A | 1.41 (1.07, 1.87) | N/A |
| Nonurban-Adjacent Rural * High Vaccine Hesitancy | N/A | N/A | N/A | 5.34 (1.69, 16.9) | N/A |
| Age Group |  |  |  |  |  |
| <30 | 0.86 (0.80, 0.92) | 0.83 (0.77, 0.89) | 0.90 (0.83, 0.97) | 0.86 (0.80, 0.92) | 0.86 (0.80, 0.92) |
| 30-49 | Reference | Reference | Reference | Reference | Reference |
| 50-64 | 0.87 (0.82, 0.92) | 0.88 (0.83, 0.93) | 0.87 (0.81, 0.93) | 0.87 (0.82, 0.92) | 0.87 (0.82, 0.92) |
| 65-75 | 0.65 (0.61, 0.70) | 0.66 (0.61, 0.70) | 0.70 (0.63, 0.72) | 0.66 (0.61, 0.70) | 0.66 (0.61, 0.70) |
| >75 | 0.58 (0.53, 0.64) | 0.59 (0.54, 0.65) | 0.62 (0.53, 0.72) | 0.59 (0.54, 0.64) | 0.60 (0.54, 0.65) |
| Gender |  |  |  |  |  |
| Female | Reference | Reference | Reference | Reference | Reference |
| Male | 1.10 (1.05, 1.15) | 1.09 (1.04, 1.14) | 1.08 (1.04, 1.13) | 1.10 (1.05, 1.14) | 1.09 (1.05, 1.14) |
| Race/Ethnicity |  |  |  |  |  |
| Non-Hispanic/Latinx White | Reference | Reference | Reference | Reference |  |
| Non-Hispanic/Latinx Black | 1.00 (0.92, 1.08) | 1.00 (0.92, 1.09) | 1.00 (0.92, 1.09) | 1.00 (0.92, 1.09) | 0.90 (0.82, 0.97) |
| Hispanic/Latinx | 1.03 (0.96, 1.10) | 1.05 (0.98, 1.13) | 0.89 (0.81, 0.96) | 1.02 (0.96, 1.10) | 0.98 (0.91, 1.05) |
| Asian American/Pacific Islander | 0.92 (0.82, 1.02) | 0.94 (0.84, 1.04) | 0.75 (0.64, 0.86) | 0.91 (0.82, 1.01) | 0.85 (0.76, 0.94) |
| Other | 1.05 (0.97, 1.15) | 1.05 (0.96, 1.15) | 0.92 (0.83, 1.02) | 1.05 (0.96, 1.15) | 0.99 (0.91, 1.08) |
| Missing/Unknown | 0.76 (0.65, 0.89) | 0.74 (0.63, 0.88) | 0.68 (0.51, 0.84) | 0.76 (0.64, 0.89) | 0.72 (0.61, 0.85) |
| Comorbid Conditions^1^ |  |  |  |  |  |
| Heart Disease | 1.01 (0.93, 1.10) | 0.98 (0.90, 1.07) | 1.04 (0.95, 1.13) | 1.01 (0.93, 1.10) | 1.02 (0.94, 1.11) |
| Peripheral Vascular Disease | 1.06 (0.98, 1.15) | 1.06 (0.98, 1.15) | 0.92 (0.84, 1.00) | 1.06 (0.98, 1.15) | 1.06 (0.98, 1.15) |
| Peptic Ulcer Disease | 0.97 (0.83, 1.14) | 0.98 (0.84, 1.16) | 1.01 (0.84, 1.17) | 0.98 (0.83, 1.14) | 0.98 (0.84, 1.15) |
| Mild or Severe Liver Disease | 0.90 (0.82, 0.97) | 0.87 (0.80, 0.95) | 0.91 (0.83, 1.00) | 0.90 (0.82, 0.97) | 0.89 (0.82, 0.97) |
| Diabetes Mellitus | 0.97 (0.91, 1.03) | 0.98 (0.92, 1.05) | 1.00 (0.94, 1.07) | 0.97 (0.92, 1.04) | 0.97 (0.91, 1.03) |
| Hemiplegia or Paraplegia | 0.95 (0.75, 1.19) | 0.96 (0.76, 1.21) | 0.94 (0.71, 1.17) | 0.95 (0.75, 1.19) | 0.94 (0.75, 1.18) |
| Stroke | 0.86 (0.78, 0.94) | 0.85 (0.77, 0.93) | 0.88 (0.78, 0.97) | 0.86 (0.78, 0.94) | 0.86 (0.79, 0.95) |
| Renal Disease | 1.17 (1.08, 1.27) | 1.15 (1.05, 1.25) | 1.14 (1.05, 1.22) | 1.17 (1.08, 1.27) | 1.19 (1.10, 1.29) |
| Any Cancer (Except Skin) | 0.87 (0.81, 0.94) | 0.87 (0.80, 0.93) | 0.86 (0.78, 0.94) | 0.87 (0.81, 0.94) | 0.87 (0.81, 0.94) |
| Documented Obesity | 1.13 (1.08, 1.18) | 1.16 (1.10, 1.21) | 1.14 (1.09, 1.19) | 1.13 (1.08, 1.18) | 1.15 (1.10, 1.21) |
| ISC (RD, SOT, MS, HIV, BMT) | 1.32 (1.24, 1.41) | 1.32 (1.24, 1.41) | 1.35 (1.29, 1.42) | 1.32 (1.24, 1.41) | 1.33 (1.24, 1.41) |
| Vaccine Manufacturer |  |  |  |  |  |
| Pfizer BioNTech | Reference | Reference | Reference | Reference | Reference |
| Moderna NIAID | 0.78 (0.73, 0.82) | 0.78 (0.74, 0.82) | 0.63 (0.57, 0.69) | 0.77 (0.73, 0.82) | 0.79 (0.75, 0.83) |
| Time Period of Second Vaccination Administration |  |  |  |  |  |
| Pre-Delta (< June 20, 2021) | Reference | Reference | Reference | Reference | Reference |
| Post-Delta (>= June 20, 2021) | 1.20 (1.10, 1.30) | 0.90 (0.84, 0.97) | 0.95 (0.86, 1.03) | 1.20 (1.10, 1.30) | 1.20 (1.11, 1.30) |
| Pre-Vaccine SARS-CoV-2 Infection History |  |  |  |  |  |
| No Reported Hx of SARS-CoV-2 | Reference | N/A | Reference | Reference | Reference |
| Reported Hx of SARS-CoV-2 | 0.25 (0.22, 0.29) | N/A | 0.20 (0.07, 0.34) | 0.25 (0.22, 0.29) | 0.25 (0.22, 0.29) |
| Region |  |  |  |  |  |
| Northeast | Reference | Reference | Reference | Reference |  |
| Midwest | 1.10 (1.01, 1.20) | 1.22 (1.12, 1.33) | 1.30 (1.06, 1.54) | 1.10 (1.01, 1.20) | 1.38 (1.27, 1.50) |
| South | 0.88 (0.80, 0.96) | 0.96 (0.87, 1.06) | 1.70 (1.45, 1.95) | 0.88 (0.80, 0.97) | 1.05 (0.96, 1.15) |
| West | 1.30 (1.20, 1.40) | 1.44 (1.33, 1.56) | 1.84 (1.65, 2.03) | 1.32 (1.22, 1.43) | 1.51 (1.40, 1.64) |
| 1. Definitions and logic used for rural categorization, vaccine hesitancy categories, Social Deprivation Index, Social Vulnerability Index, county vaccination rates, and comorbid conditions provided in eMethods S-1. | | | | | |

**S6 Table. Multivariable Cox-Proportional Hazard Ratios for SARS-CoV-2 Infection in Unvaccinated or Undocumented Vaccination Status Adults, January 1, 2021 – December 20, 2021**

| **Covariate** | **Hazard Ratio**  **(95% Confidence Interval)** | ***p* value** |
| --- | --- | --- |
| Rurality^1^ |  |  |
| Urban | Reference |  |
| Urban-Adjacent Rural | 1.03 (1.02, 1.04) | <0.001 |
| Nonurban-Adjacent Rural | 1.04 (1.02, 1.06) | <0.001 |
| Vaccine Hesitancy Category^1^ |  |  |
| Low (≤ 5% hesitant) | Reference |  |
| Medium (6-15% hesitant) | 1.26 (1.25, 1.28) | <0.001 |
| High (>15% hesitant) | 1.27 (1.25, 1.29) | <0.001 |
| County Vaccination Rates^1^ |  |  |
| Low (<70% vaccinated) | 1.09 (1.08, 1.10) | <0.001 |
| Medium (70-77% vaccinated) | 1.01 (1.00, 1.02) | 0.3 |
| High (>77% vaccinated) | Reference |  |
| Age Group |  |  |
| <30 | 1.02 (1.01, 1.03) | <0.001 |
| 30-49 | Reference |  |
| 50-64 | 0.87 (0.86, 0.87) | <0.001 |
| 65-75 | 0.76 (0.75, 0.77) | <0.001 |
| >75 | 0.84 (0.83, 0.86) | <0.001 |
| Gender |  |  |
| Female | Reference |  |
| Male | 1.15 (1.14, 1.15) | <0.001 |
| Race/Ethnicity |  |  |
| Non-Hispanic/Latinx White | Reference |  |
| Non-Hispanic/Latinx Black | 1.07 (1.06, 1.08) | <0.001 |
| Hispanic/Latinx | 1.22 (1.20, 1.23) | <0.001 |
| Asian American/Pacific Islander | 1.03 (1.00, 1.05) | 0.026 |
| Other | 1.06 (1.05, 1.07) | <0.001 |
| Missing/Unknown | 1.67 (1.65, 1.70) | <0.001 |
| Comorbid Conditions^1^ |  |  |
| Heart Disease | 0.86 (0.85, 0.88) | <0.001 |
| Peripheral Vascular Disease | 0.86 (0.85, 0.87) | <0.001 |
| Peptic Ulcer Disease | 0.80 (0.78, 0.83) | <0.001 |
| Mild or Severe Liver Disease | 0.84 (0.83, 0.85) | <0.001 |
| Diabetes Mellitus | 0.98 (0.97, 0.99) | <0.001 |
| Hemiplegia or Paraplegia | 0.79 (0.76, 0.81) | <0.001 |
| Stroke | 0.84 (0.82, 0.85) | <0.001 |
| Renal Disease | 1.02 (1.01, 1.04) | 0.003 |
| Any Cancer (Except Skin) | 0.63 (0.62, 0.64) | <0.001 |
| Documented Obesity | 1.02 (1.01, 1.02) | <0.001 |
| ISC (RD, SOT, MS, HIV, BMT) | 0.89 (0.88, 0.90) | <0.001 |
| Region |  |  |
| Northeast | Reference |  |
| Midwest | 0.88 (0.87, 0.90) | <0.001 |
| South | 0.91 (0.90, 0.93) | <0.001 |
| West | 1.00 (0.99, 1.01) | >0.9 |
| 1. Definitions and logic used for rural categorization, vaccine hesitancy categories, Social Deprivation Index, county vaccination rates, and comorbid conditions provided in eMethods S-1. | | |

**S7 Table. Univariable Cox-Proportional Hazard Ratios for SARS-CoV-2 Infection in Unvaccinated or Undocumented Vaccination Status Adults, January 1, 2021 – December 20, 2021**

| **Covariate** | **Hazard Ratio**  **(95% Confidence Interval)** | ***p* value** |
| --- | --- | --- |
| Rurality^1^ |  |  |
| Urban | Reference |  |
| Urban-Adjacent Rural | 1.04 (1.03, 1.05) | <0.001 |
| Nonurban-Adjacent Rural | 1.03 (1.01, 1.05) | <0.001 |
| Vaccine Hesitancy Category^1^ |  |  |
| Low (≤ 5% hesitant) | Reference |  |
| Medium (6-15% hesitant) | 1.29 (1.28, 1.30) | <0.001 |
| High (>15% hesitant) | 1.26 (1.25, 1.27) | <0.001 |
| County Vaccination Rates^1^ |  |  |
| Low (<70% vaccinated) | 1.08 (1.07, 1.10) | <0.001 |
| Medium (70-77% vaccinated) | 0.98 (0.97, 0.99) | 0.002 |
| High (>77% vaccinated) | Reference |  |
| Age Group |  |  |
| <30 | 1.06 (1.05, 1.07) | <0.001 |
| 30-49 | Reference |  |
| 50-64 | 0.80 (0.79, 0.81) | <0.001 |
| 65-75 | 0.64 (0.63, 0.65) | <0.001 |
| >75 | 0.65 (0.64, 0.66) | <0.001 |
| Gender |  |  |
| Female | Reference |  |
| Male | 1.07 (1.07, 1.08) | <0.001 |
| Race/Ethnicity |  |  |
| Non-Hispanic/Latinx White | Reference |  |
| Non-Hispanic/Latinx Black | 1.07 (1.06, 1.09) | <0.001 |
| Hispanic/Latinx | 1.34 (1.32, 1.35) | <0.001 |
| Asian American/Pacific Islander | 1.00 (0.98, 1.02) | 0.9 |
| Other | 1.08 (1.06, 1.09) | <0.001 |
| Missing/Unknown | 1.84 (1.80, 1.87) | <0.001 |
| Comorbid Conditions^1^ |  |  |
| Heart Disease | 0.69 (0.68, 0.70) | <0.001 |
| Peripheral Vascular Disease | 0.65 (0.64, 0.66) | <0.001 |
| Peptic Ulcer Disease | 0.67 (0.65, 0.69) | <0.001 |
| Mild or Severe Liver Disease | 0.73 (0.72, 0.74) | <0.001 |
| Diabetes Mellitus | 0.83 (0.82, 0.83) | <0.001 |
| Hemiplegia or Paraplegia | 0.64 (0.62, 0.66) | <0.001 |
| Stroke | 0.65 (0.64, 0.66) | <0.001 |
| Renal Disease | 0.76 (0.75, 0.77) | <0.001 |
| Any Cancer (Except Skin) | 0.53 (0.53, 0.54) | <0.001 |
| Documented Obesity | 1.00 (1.00, 1.01) | 0.2 |
| ISC (RD, SOT, MS, HIV, BMT) | 0.78 (0.77, 0.79) | <0.001 |
| Region |  |  |
| Northeast | Reference |  |
| Midwest | 0.94 (0.92, 0.95) | <0.001 |
| South | 0.96 (0.94, 0.97) | <0.001 |
| West | 1.08 (1.06, 1.09) | <0.001 |
| 1. Definitions and logic used for rural categorization, vaccine hesitancy categories, Social Deprivation Index, county vaccination rates, and comorbid conditions provided in eMethods S-1. | | |

**S8 Table. Multivariable Odds Ratios for 30-Day Hospitalization or Adverse Event Following SARS-CoV-2 Infection in Persons Vaccinated and Unvaccinated or without Documented Vaccination Event, January 2021 - December 2021**

| **Covariate** | **Outcome 1: 30-Day Hospitalization Following SARS-CoV-2 Infection**  **Odds Ratio (95% Confidence Interval) ^1^** | **Outcome 2: 30-Day Adverse Event Following SARS-CoV-2 Infection**  **Odds Ratio (95% Confidence Interval) ^1^** |
| --- | --- | --- |
| Vaccination Status |  |  |
| Vaccinated | 0.58 (0.55, 0.62) | 0.38 (0.30, 0.46) |
| Unvaccinated or Undocumented  Vaccination Status | Reference | Reference |
| Rurality^2^ |  |  |
| Urban | Reference | Reference |
| Urban-Adjacent Rural | 1.12 (1.07, 1.17) | 1.28 (1.14, 1.42) |
| Nonurban-Adjacent Rural | 1.10 (1.08, 1.13) | 1.24 (1.16, 1.32) |
| Vaccine Hesitancy Category^2^ |  |  |
| Low (≤ 5% hesitant) | Reference | Reference |
| Medium (6-15% hesitant) | 1.11 (1.07, 1.14) | 0.93 (0.85, 1.02) |
| High (>15% hesitant) | 1.11 (1.07, 1.14) | 1.12 (1.02, 1.23) |
| County Vaccination Rates^2^ |  |  |
| Low (<70%) | 1.31 (1.26, 1.35) | 1.45 (1.31, 1.61) |
| Medium (70-77%) | 1.08 (1.05, 1.12) | 1.27 (1.16, 1.40) |
| High (>77%) | Reference | Reference |
| Age Group |  |  |
| <30 | 1.12 (1.09, 1.14) | 0.44 (0.38, 0.51) |
| 30-49 | Reference | Reference |
| 50-64 | 0.91 (0.89, 0.93) | 2.02 (1.88, 2.18) |
| 65-75 | 1.04 (1.01, 1.07) | 3.16 (2.92, 3.42) |
| >75 | 1.45 (1.39, 1.50) | 6.14 (5.65, 6.67) |
| Gender |  |  |
| Female | Reference | Reference |
| Male | 1.03 (1.01, 1.05) | 1.45 (1.38, 1.52) |
| Race/Ethnicity |  |  |
| Non-Hispanic/Latinx White | Reference | Reference |
| Non-Hispanic/Latinx Black | 2.61 (2.55, 2.67) | 1.31 (1.22, 1.40) |
| Hispanic/Latinx | 1.58 (1.54, 1.62) | 1.44 (1.33, 1.56) |
| Asian American/Pacific Islander | 1.17 (1.11, 1.24) | 1.73 (1.49, 2.00) |
| Other | 1.18 (1.14, 1.22) | 1.39 (1.26, 1.53) |
| Missing/Unknown | 0.37 (0.35, 0.40) | 1.39 (1.19, 1.61) |
| Comorbid Conditions^2^ |  |  |
| Heart Disease | 1.49 (1.45, 1.54) | 2.30 (2.17, 2.43) |
| Peripheral Vascular Disease | 1.31 (1.27, 1.36) | 1.10 (1.03, 1.17) |
| Peptic Ulcer Disease | 1.30 (1.23, 1.38) | 1.22 (1.08, 1.36) |
| Mild or Severe Liver Disease | 1.39 (1.35, 1.43) | 2.26 (2.13, 2.40) |
| Diabetes Mellitus | 1.28 (1.25, 1.31) | 2.38 (2.25, 2.50) |
| Hemiplegia or Paraplegia | 1.66 (1.54, 1.79) | 2.21 (1.97, 2.48) |
| Stroke | 1.29 (1.25, 1.34) | 1.36 (1.28, 1.45) |
| Renal Disease | 1.29 (1.25, 1.33) | 1.55 (1.46, 1.64) |
| Any Cancer (Except Skin) | 1.09 (1.06, 1.13) | 1.19 (1.11, 1.26) |
| Documented Obesity | 1.20 (1.18, 1.22) | 1.14 (1.09, 1.20) |
| ISC (RD, SOT, MS, HIV, BMT) | 1.06 (1.03, 1.09) | 0.99 (0.93, 1.06) |
| Prior SARS-CoV-2 Infection |  |  |
| No Reported Hx of SARS-CoV-2 | Reference | Reference |
| Reported Hx of SARS-CoV-2 | 0.62 (0.59, 0.65) | 0.80 (0.71, 0.89) |
| Region |  |  |
| Northeast | Reference | Reference |
| Midwest | 1.10 (1.06, 1.14) | 1.27 (1.14, 1.42) |
| South | 1.41 (1.35, 1.46) | 1.15 (1.03, 1.29) |
| West | 1.19 (1.14, 1.23) | 1.06 (0.95, 1.19) |
| 1. Severity categories following SARS-CoV-2 infection classified as outpatient-only visit (no hospitalization within 30 days of diagnosis), COVID-associated hospitalization (within 30 days of diagnosis), COVID-19-associated adverse event (mechanical ventilation, ECMO, or death or transfer to hospice within 30 days of diagnosis).  2. Definitions and logic used for rural categorization, vaccine hesitancy categories, county vaccination rates, and comorbid conditions provided in eMethods S-1. | | |

**S1 Methods. National COVID Cohort Collaborative (N3C) Methods**

This supplementary methods section describes the data collection strategy, concept set definitions, and overall structure of the N3C Data Enclave. Its contents are adapted from previous published work developed by the Immunosuppressed/Compromised Domain Team: <https://covid.cd2h.org/compromised>.

**Data Access**

Researchers request access to either a HIPAA Limited Data Set (LDS) or a HIPAA Safe Harbor (de-identified) version of the N3C data through a project-specific Data Use Request. The de-identified data set is generated from the LDS data by shifting all dates up to 180 days (except the birth year) and reducing zip codes to 3 digits.  N3C represents the largest centralized enclave of LDS captured in the United States for clinical research, relying on standardized robustness checks to ensure high fidelity data. Once approved, access to N3C data is achieved through a secure Enclave, which is a virtual, secure, cloud-based environment that meets federal data security standards. Technical measures have been put in place such that no patient-level data may be downloaded or removed from the environment, and all summarized data (including images) must be approved by NCATS prior to publication or distribution.[1]

**N3C Phenotype**

The N3C Phenotype is a broad set of criteria to identify patients important for COVID-19 research and for inclusion in the N3C database. This includes all lab-confirmed, suspected, and possible cases of COVID-19, and a demographically matched (on age group, sex, race, and ethnicity) control group who tested negative for COVID-19 at a ratio of 1:2 (cases: controls).  Briefly, COVID-19 cases are defined as any patient with an encounter after 1/1/2020 with either 1) one or more positive COVID-19 lab tests, 2) one or more “strong positive” diagnosis codes, or 3) two or more “weak positive” diagnosis codes during the same encounter on or prior to 5/1/2020.  Control cases must 1) not qualify as a case, 2) match a case demographically, 3) have one or more non-positive COVID-19 lab tests after 1/1/2020, and 4) have at least 10 days between the minimum and maximum encounter date to eliminate person’s that were only seen for testing. ^2^ The majority of COVID-19 positivity is from PCR or Antigen tests (78%) or physician diagnosis (19%) with the remaining coming from antibody testing (3%). Additional details, including specific labs and diagnostic codes can be found in the N3C Phenotype GitHub repository (<https://github.com/National-COVID-Cohort-Collaborative/Phenotype_Data_Acquisition/wiki/Latest-Phenotype>).[2]

**Data Characteristics**

N3C data is both retrospective and prospective.  Retrospective records dating back to 1/1/2018 are included for historical medical context for all individuals in N3C.  In addition, once a patient is added to N3C (case or control), they remain in the cohort with new records being added until they qualify as a COVID-19 case.  If a control qualifies as a case a new demographically matched control is selected and historical records are retrieved back to 1/1/2018 for inclusion in N3C. Patient records include a variety of clinical data that is modeled by the OMOP CDM, including demographics, encounters, diagnosis, procedures, medications, vitals, labs, vaccinations and more.  Additional details on the OMOP CDM can be found on the OHDSI (Observational Health Data Sciences and Informatics) website [<https://www.ohdsi.org/data-standardization/the-common-data-model/>] or OMOP GitHub repository [<https://ohdsi.github.io/CommonDataModel/>].[3]

**Experimental Design and Data Analysis**

Designing experiments and performing data analysis must be done entirely within the N3C Enclave.  Computational phenotypes to identify cohorts can only be designed using concepts defined by OMOP and available in the N3C Enclave as researchers do not have access to the original EMR record.  As data is ingested from dozens of data partners using different CDMs some data that would normally be available in the original EHR, and used in clinically validated computational phenotypes, is either not present (e.g. O2 saturation) or sparsely populated (e.g. BMI).  Thus, N3C computational phenotypes and data analyses must take this into account during the experimental design phase and recognize that these may be possible limitations to the analysis.^3^

**N3C Cohort Definition Process**

Defining a cohort in N3C first requires a computational phenotype be developed that utilizes Concepts and Concept Sets from the OMOP Standard Vocabulary.  During harmonization, source vocabularies like ICD-10 and RxNorm are mapped to the OMOP Standard Vocabulary.  A computational phenotype is developed by identifying those diagnoses, labs, medications, and other criteria using source vocabularies.  Then the ATLAS tool (<http://atlas-demo.ohdsi.org/>) can be used to map those to OMOP Concepts.  Once all relevant OMOP Concepts are collected they can be grouped into Concept Sets, which is just a list of one or more related Concepts.  For example, Supplementary Method Table 1 provides an example where the concept of wheelchair dependence is first defined using source vocabularies, then is mapped to OMOP Concepts followed by the creation of a custom Concept Set that is imported into the N3C Concept Set table.  Once imported, Concept IDs and Concept Sets can be used to identify patients matching specific criteria to create a cohort.

| Source Vocabulary | Source ID | OMOP Concept ID | OMOP Domain | N3CConcept Set Name | N3C Concept Set ID |
| --- | --- | --- | --- | --- | --- |
| CPT4 | 97542 | 2314299 | Procedure | [N3C][ISC] Wheelchair Dependence (v1) | 1869047 |
| ICD10-CM | Z99.3 | 45547382 | Condition |  |  |

**Supplementary Methods Table 1:** Example of how a source vocabulary term is mapped to a OMOP Concept Set.  This specific concept set actually has 246 source vocabulary terms and can be accessed on ATLAS at <http://atlas-demo.ohdsi.org/#/conceptset/1869047/expression>.

**N3C Data Partners**

As of March 23, 2022 (N3C Release 71), N3C harmonizes EHR data from a data partner network that includes 72 institutions from across the United States. The majority of N3C data partners represent tertiary care centers (Supplementary Methods Table 1).  An additional 34 sites have signed Data Transfer Agreements with their data pending for availability (<https://covid.cd2h.org/dashboard/>).  Supplementary Table 2 shows the current list of N3C data partners.

**Data Partner Study Inclusion Criteria**

We included persons in this study based on N3C data partner reporting practices. Our primary data partner requirements were vaccination reporting (>10% of patient population with a documented vaccination), 5-digit ZIP Code availability, condition reporting, and data latency (most recent data contribution ≥ December 20, 2021). N3C data partners report varying levels of vaccination information, likely a result of a presence or lack of integration with state vaccine registries or health information exchanges. To account for this, we include only those sites with higher baseline vaccination reporting on patients including in their N3C transmission to reduce contamination with unlabeled vaccinated persons in our unvaccinated cohort.

**eFigure S-1** provides a profile of data partners included in this study by vaccination availability and rural distribution. This follows a similar approach used by the four source data models that all rely on minimum reporting requirements for site inclusion into network studies [4-7]. We also provide an overview of data partner reporting availability, including earliest vaccination reporting, latest vaccination reporting, and latest data available in the N3C Enclave in **eFigure S-2**. Our patient cohort includes adults (>17 years) who received two mRNA COVID-19 vaccinations between January 1, 2021, and September 21, 2021. Persons with missing age or gender were excluded, as documented in **eFigure S-3**.

**Definitions (Computable Phenotypes)**

Coding algorithms used to identify cohorts in the N3C repository utilize OMOP Concept Identifiers (Concept IDs) that are associated with a patient’s submitted health records, which includes reported conditions, drugs, procedures, lab results and more. Concept IDs map a variety of medical vocabularies reported from each submitting institution, such as ICD10, CPT, RxNorm, SNOMED, and more, to a single unique identifier. Groups of related Concept IDs form a Concept Set, which can be used to query the N3C repository for patients meeting certain criteria. In this work we developed a series of OMOP Concept Sets along with inclusion/exclusion logic to define our cohorts.

All codesets used to define COVID-19 positivity, covariates, and outcomes of interest are provided below. All Concept IDs and Concept Sets listed in the supplementary information for this work can be searched for specific source vocabulary terms, such as ICD10 and RxNorm, using the ATLAS demo browser (<http://atlas-demo.ohdsi.org/>). had data released for analysis and the N3C Enclave, and (3) had data that passed initial quality checks. Definitions include systematic utilization of both source mappings to OMOP standards done during the data ingestion process and mapping to source concepts for certain concepts that less clearly structured when transmitted to N3C.

**Concept Sets Definitions. Key variables and concept definitions**

| **Variable** |  | **Concepts and Logic** |
| --- | --- | --- |
|  | ***Primary Cohort Definition*** | |
| ***SARS-CoV-2 Infection*** |  | **Concept Set Names (latest codeset ID) with Description:**  N3C Covid diagnosis (35486128)  *Description: Corresponds to ICD10CM Code: U07.1 (condition). Includes 1 Concept (Disease caused by 2019-nCoV - Concept Id: 840539006).*  ATLAS SARS-CoV-2 rt-PCR and Ag (651620200)  *Description: Includes 55 Concepts (Measurements), characterizing a positive PCR or Antigen test.*  Atlas #818 [N3C] CovidAntibody retry (45478367)  *Description: Includes 24 Concepts (Measurements), characterizing a positive Antibody test.*  ResultsPos (400691529)  *Description: Includes 6 Concepts , collecting all the affirmative measurement results*  **Logic:**  Patient record must be associated with a positive SARS-CoV-2 lab test. The above concept sets are part of the N3C inclusion criteria for data submissions sent to N3C. This analysis relies on a more restrictive definition, only utilizing positive PCR or Ag test result observed after the introduction of vaccinations in the United States. Antibody-only positive results were excluded after December 31, 2020, to account for broad distribution of the COVID-19 vaccination in the general population. |
|  | ***Key Community Factors*** | |
| Rurality |  | **Logic:**  Patients were included if they had a 5-digit ZIP Code, which was mapped to RUCA Codes through an external dataset maintained by the USDA Economic Research Services: <https://www.ers.usda.gov/data-products/rural-urban-commuting-area-codes/>. This dataset is available in N3C here: [[EXTDATASET-59] RUCA Rural-Urban Commuting Area Codes 1](https://unite.nih.gov/workspace/compass/view/ri.compass.main.folder.ade189b0-39fe-45b8-943c-fb4cf38fb996). Patients were mapped to three categories based on this ZIP Code crosswalk as follows based on primary RUCA Code designation:  Urban:   \| 1.  Metropolitan area core: primary flow within an urbanized area (UA) \| \| --- \| \| 2.   Metropolitan area high commuting: primary flow 30% or more to a UA \| \| 3.  Metropolitan area low commuting: primary flow 10% to 30% to a UA \|   Urban-Adjacent Rural:   \| 4. Micropolitan area core: primary flow within an Urban Cluster of 10,000 to 49,999 (large UC) \| \| \| --- \| --- \| \| 5. Micropolitan high commuting: primary flow 30% or more to a large UC \| \| \| 7. Small town core: primary flow within an Urban Cluster of 2,500 to 9,999 (small UC) \| \| 8. Small town high commuting: primary flow 30% or more to a small UC \|   Nonurban-Adjacent Rural:   \| 6. Micropolitan low commuting: primary flow 10% to 30% to a large UC \| \| --- \| \| 9. Small town low commuting: primary flow 10% to 30% to a small UC \| \| 10.  Rural areas: primary flow to a tract outside a UA or UC \| |
| Vaccine Hesitancy |  | **Logic:**  Patients were included if they had a 5-digit ZIP Code, which was mapped to [Delphi's COVID-19 Trends and Impact Surveys (CTIS)](https://delphi.cmu.edu/covid19/ctis/). This survey has been active since April 2020, and it utilizes data collected through Facebook representing around 50,000 daily participants engaged in instrument collection. Survey results used were from an October 1, 2021 release, which is available here: <https://www.healthdata.org/sites/default/files/files/Projects/COVID/2021/cleaned_vaccine_10.01.2021.csv>. Patients were categories into three levels based on degree of vaccine hesitancy in their residential ZIP Code:  1) Low ZIP Code Vaccine Hesitancy (≤5% of respondents were vaccine hesitant)  2) Medium ZIP Code Vaccine Hesitancy (6-15% of respondents were vaccine hesitant)  3) High ZIP Code Vaccine Hesitancy (>15% of respondents were vaccine hesitant) |
| County Vaccination Rates |  | **Logic:**  Patient county of residence, when available, was mapped to the [CDC vaccination reporting for US counties](https://www.cdc.gov/coronavirus/2019-ncov/vaccines/distributing/about-vaccine-data.html). When counties were not available, 5-digit ZIP Codes were mapped to county of residence using the [HUD ZIP-Code-to-County crosswalk for 2010](https://www.huduser.gov/portal/datasets/usps_crosswalk.html). CDC vaccination data was extracted from public health datasets through September 21, 2021: <https://data.cdc.gov/Vaccinations/COVID-19-Vaccinations-in-the-United-States-County/8xkx-amqh>. Patients were categorized into three levels based on county vaccination rates through this period as follows:  1) Low county vaccination rate (<70% adult vaccination documented through September 21, 2021)  2) Medium county vaccination rate (70-77% adult vaccination documented through September 21, 2021)  3) High county vaccination rate (>77% adult vaccination documented through September 21, 2021) |
| ***Sensitivity Analysis Key Comparison*** | | |
| Social Vulnerability Index |  | **Logic:**  Patient county of residence, when available, was mapped to the [CDC/ATSDR Social Vulnerability Index (SVI)](https://www.atsdr.cdc.gov/placeandhealth/svi/index.html). When counties were not available, 5-digit ZIP Codes were mapped to county of residence using the [HUD ZIP-Code-to-County crosswalk for 2010](https://www.huduser.gov/portal/datasets/usps_crosswalk.html). SVI public-use datasets are available here: <https://www.atsdr.cdc.gov/placeandhealth/svi/data_documentation_download.html>. SVI is a percentile indicator ranging from 0 to 1, with higher values indicating greater social vulnerability. Patients were categorized into three levels based on county of residence SVI:  1) Low SVI (<0.334)  2) Medium SVI ((0.334-0.666)  3) High SVI (>0.666) |
|  | ***COVID-19 Disease Severity*** | |
| Hospitalization |  | **Visit Concept IDs and Names:**  262 Emergency Room and Inpatient Visit  8717 Inpatient Hospital  9201 Inpatient Visit  581379 Inpatient Critical Care Facility  **Logic:**  No concept sets were used in this definition. Includes patients with a visit start date between 14 days prior to the earliest covid diagnosis to 30 days after covid diagnosis that are also associated with one of the listed visit concept IDs. |
| Invasive mechanical ventilation |  | **Concept Set Names (latest codeset ID):**  Invasive Mechanical Ventilation 2OCT20 (179437741)  **Logic:**  Includes patients associated with any procedure codes in the concept sets listed that had a procedure date during their initial post-COVID hospitalization. |
| ECMO |  | **Concept Set Names (latest codeset ID):**  Kostka – ECMO (415149730)  **Logic:**  Includes patients associated with any procedure codes in the concept sets listed that had a procedure date during their initial post-COVID hospitalization. |
|  |  |  |
| Death or Transfer to Hospice |  | **Logic:**  No concepts or concept sets were used.  Patients were flagged as deceased if a valid entry with a date was included in the death table, which is part of the OMOP data model used in the Enclave. Patients who discharged to hospice (OMOP concept IDs 8546, 32225, 32226) were included as well, with their transfer date approximating a date of death for modeling purposes. |
|  | ***Comorbid Conditions*** | |
| Severe cardiovascular event (Congestive heart failure and myocardial infarction) |  | **Concept Set Names (latest codeset ID):**  Charlson - CHF (359043664)  Charlson - MI (259495957)  **Logic:**  Includes patients associated with any condition codes in the concept sets listed with an occurrence date before the earliest covid diagnosis. |
| Peripheral vascular diseases |  | **Concept Set Names (latest codeset ID):**  Charlson - PVD (376881697)  **Logic:**  Includes patients associated with any condition codes in the concept sets listed with an occurrence date before the earliest covid diagnosis. |
| Stroke |  | **Concept Set Names (latest codeset ID):**  Charlson - Stroke (652711186)  **Logic:**  Includes patients associated with any condition codes in the concept sets listed with an occurrence date before the earliest covid diagnosis. |
| Dementia |  | **Concept Set Names (latest codeset ID):**  Charlson - Dementia (78746470)  **Logic:**  Includes patients associated with any condition codes in the concept sets listed with an occurrence date before the earliest covid diagnosis. |
| Pulmonary Diseases |  | **Concept Set Names (latest codeset ID):**  Charlson - Pulmonary (514953976)  **Logic:**  Includes patients associated with any condition codes in the concept sets listed with an occurrence date before the earliest covid diagnosis. |
| Peptic ulcer diseases |  | **Concept Set Names (latest codeset ID):**  Charlson - PUD (510748896)  **Logic:**  Includes patients associated with any condition codes in the concept sets listed with an occurrence date before the earliest covid diagnosis. |
| Liver diseases (mild and severe liver diseases) |  | **Concept Set Names (latest codeset ID):**  Charlson - LiverMild (494981955)  Charlson - LiverSevere (248333963)  **Logic:**  Includes patients associated with any condition codes in the concept sets listed with an occurrence date before the earliest covid diagnosis. |
| Diabetes mellitus (diabetes mellitus and diabetes mellitus with complications) |  | **Concept Set Names (latest codeset ID):**  Charlson - DM (719585646)  Charlson - DMcx (403438288)  **Logic:**  Includes patients associated with any condition codes in the concept sets listed with an occurrence date before the earliest covid diagnosis. |
| Renal diseases |  | **Concept Set Names (latest codeset ID):**  Charlson - Renal (220495690)  **Logic:**  Includes patients associated with any condition codes in the concept sets listed with an occurrence date before the earliest covid diagnosis. |
| Cancer (metastatic and non-metastatic) |  | **Concept Set Names (latest codeset ID):**  Charlson - Cancer (535274723)  Charlson - Mets (378462283)  **Logic:**  Includes patients associated with any condition codes in the concept sets listed with an occurrence date before the earliest covid diagnosis. |
|  |  | ***Additional Comorbid Conditions (Non-Charlson Comorbidity Index)*** |
| Documented Obesity |  | **Concept Set Names (latest codeset ID):**  Concepts for obesity from Observation Domain, Concept Name (concept ID):  Body mass index 30+ - obesity (4060985)  Body mass index 40+ - severely obese (4256640)  Obese (4215968)  Obese class I (763588)  Obese class II (763589)  Obese class III (37311728)  **Logic:**  Includes patients with reported height and weight or calculated BMI with most proximal measurement information prior to index date for calculated BMI greater than or equal to 30.0. If no measurement information is available, documented obesity observations are used to establish pre-vaccination or pre-SARS-CoV-2 infection obesity status. |
| HIV infection |  | **Concept Set Names (latest codeset ID):**  HIV drugs (331061593)  hiv infection (382527336)  cs_isc_hiv_labs_procedures (684826767)  PrEP Concept IDs and Names:  emtricitabine 100 MG / tenofovir disoproxil fumarate 150 MG Oral Tablet [Truvada] (35606583)  emtricitabine 200 MG / tenofovir disoproxil fumarate 300 MG Oral Tablet [Truvada] (1710316 )  emtricitabine 167 MG / tenofovir disoproxil fumarate 250 MG Oral Tablet [Truvada] (35606591)  emtricitabine 200 MG / tenofovir alafenamide 25 MG Oral Tablet (35604225)  emtricitabine 133 MG / tenofovir disoproxil fumarate 200 MG Oral Tablet (35606585)  emtricitabine 100 MG / tenofovir disoproxil fumarate 150 MG Oral Tablet (35606581)  emtricitabine 200 MG / tenofovir disoproxil fumarate 300 MG Oral Tablet (1703093)  emtricitabine 200 MG / tenofovir alafenamide 25 MG Oral Tablet [Descovy] (35604229)  **Logic:**  Included in cohort if patient is associated with at least one of the concept sets listed in either the condition, procedures, or drugs tables. Patients only on PrEP were excluded. |
| Solid Organ Transplant |  | **Concept Set Names (latest codeset ID):**  [N3C] [ISC] Kidney Transplant Rejection (25243076)  [N3C] [ISC] Heart Transplant Rejection (610701999)  [N3C] [ISC] Liver Transplant Rejection (184979218)  [N3C] [ISC] Kidney Transplant Failure (562737056)  [N3C] [ISC] Lung Transplant Rejection (784946326)  [N3C] [ISC] Heart Transplant Failure (708583955)  [N3C] [ISC] Liver Transplant Failure (663753516)  [N3C] [ISC] Lung Transplant Failure (818309036)  [N3C] [ISC] Kidney Transplant (21035617)  [N3C] [ISC] Heart Transplant (976928531)  [N3C] [ISC] Liver Transplant (204996696)  [N3C] [ISC] Lung Transplant (402177099)  [N3C] [ISC] Pancreas Transplant (788127890)  **Logic:**  All patients associated with at least one of the listed concept sets, including records from the observation, procedure occurrence, and condition occurrence tables. |
| Multiple Sclerosis |  | **Concept Set Names (latest codeset ID):**  MS Condition Occurrence:  [N3C] [ISC] Multiple Sclerosis Condition (488007883)  MS-Specific Drug Exposure:  monomethyl fumarate (345167675)  peginterferon beta-1a (443428466)  interferonbeta-1a (359012050)  dimethylfumarate (691161549)  fingolimod (892752677)  ozanimod (49326182)  siponimod (331845926)  teriflunomide (498358747)  ocrelizumab (912320071)  glatiramer acetate (580493810)  diroximel fumarate (241307366)  interferonbeta-1b (531467540)  Non-MS-Specific Drug Exposure:  alemtuzumab (610593460)  cladribine (579114847)  natalizumab (337060277)  rituximab (287668855)  ofatumumab (141560311)  **Logic:**  A Patient is included in the MS Cohort IF one of the following holds true:  1. A patient has 2 or more visit encounters with an MS Condition Occurrence.  2. A patient has 1 visit with an MS Condition Occurrence AND has a history of exposure to a Non-MS-Specific Drug.  3. A patient has a history of exposure to at least one MS-Specific Drug. |
| Bone Marrow Transplant |  | **Concepts in Use – Name (SNOMED CT Ancestor):**  Acute rejection of bone marrow transplant (4161782)  Allograft of bone marrow from unmatched unrelated donor (44793170)  Disorder related to bone marrow transplantation (4323781)  Bone marrow transplant rejection (4125659)  Accelerated rejection of bone marrow transplant (4328283)  Allogeneic related bone marrow transplant (4125487)  Acute graft-versus-host reaction following bone marrow transplant (760855)  Lymphoproliferative disorder after transplantation of bone marrow (37018894) Disorder of transplanted bone marrow (437169)  Allogeneic unrelated bone marrow transplant (4125488)  Autologous bone marrow transplant with purging (4186582)  T-cell depleted allogeneic bone marrow graft (4122920)  Hematopoietic progenitor cell (HPC); autologous transplantation (2108457)  Autologous bone marrow transplant without purging (4059885)  Syngeneic bone marrow transplant (4121104)  Allograft of bone marrow from sibling donor (4142405)  Transplantation of bone marrow (4028623)  Disease relapse in transplant marrow (4125660)  Bone marrow transplant failure (4121274)  Growth hormone deficiency after bone marrow transplant (4182094)  Short stature associated with bone marrow transplant (4325860)  Allogeneic bone marrow transplantation with purging (40486968)  Autologous bone marrow transplant (4240337)  Chronic graft versus host disease after transplantation of bone marrow (37016153) Allogeneic bone marrow transplantation without purging (40492289)  Grafting of bone marrow using allograft from unmatched unrelated donor (40484034)  Bone marrow transplant present (42537745)  Hematopoietic progenitor cell (HPC); allogeneic transplantation per donor (2108456)  Allograft of bone marrow from haploidentical donor (44790154)  Hyperacute rejection of bone marrow transplant (4197185)  Allogeneic bone marrow transplantation (4242257)  Chronic rejection of bone marrow transplant (4332092)  Allogeneic lymphocyte infusions (2108458)  Imperfect T-cell depleted allogeneic bone marrow graft (4125486)  Hematopoietic progenitor cell (HPC); HPC boost (43527933)  Allograft of bone marrow from matched unrelated donor (4144882)  **Logic:**  Included in cohort if patient is associated with at least one of the concepts listed in either the condition, procedure, or observation tables, or the concept’s source value was one of the ICD codes Z94.81 or Z48.290. |
| Rheumatic Diseases |  | **Concepts in Use:**  Rheumatoid Arthritis: SNOMED CT Ancestor: Rheumatoid Arthritis (69896004), Seropositive Rheumatoid Arthritis (239791005) ICD-10-CM: Rheumatoid Arthritis (M05*), Seropositive Rheumatoid Arthritis (M06*)  Spondyloarthritis: SNOMED CT Ancestor: Spondyloarthritis (784332006) ICD-10-CM: Ankylosing spondylitis (M45*), Other Inflammatory Spondylopathies (M46*), Arthropathic Psoriasis (L40.5*), Enteropathic Arthropathies (M07*), Postdysenteric Arthropathy (M02.1*), Reiter's Disease (M02.3*), Other Reactive Arthropathies (M02.8), Reactive arthropathy, unspecified (M02.9)  Gout: SNOMED CT Ancestor: Gout (90560007) ICD-10-CM: Gout (M10*), Chronic Gout (M1A*)  Systemic Lupus Erythematosus: SNOMED CT Ancestor: Systemic Lupus Erythematosus (55464009) ICD-10-CM: Systemic Lupus Erythematosus (M32*)  Polymyalgia Rheumatica: SNOMED CT Ancestor: Polymyalgia Rheumatica (65323003) ICD-10-CM: Polymyalgia Rheumatica (M35.3*) and Giant Cell Arteritis with Polymyalgia Rheumatica (M31.5*)  Systemic Sclerosis: SNOMED CT Ancestor: Spondyloarthritis (89155008) ICD-10-CM: Spondyloarthritis (M34*)  Polymyositis: SNOMED CT Ancestor: Polymyositis (31384009) ICD-10-CM: Polymyositis (M33.2*)  Rheumatoid Lung Disease: SNOMED CT Ancestor: Rheumatoid Lung Disease (398726004) ICD-10-CM: Rheumatoid Lung Disease (M05.1*)  **Logic:**  SNOMED CT codes represent the standard condition mapping for the OMOP CDM. We included mappings in this standard as well as additional concepts mapped from source system data provided in ICD10 CM. Our cohort included patients with Rheumatoid Arthritis. Patients with other Rheumatic Disease were excluded from our analyses. |

**S2 Methods. List of Data Partners with Released or Pending Data in N3C**

| **Data Partner Name** | **Type** | **Data Model** | **Data Status** |
| --- | --- | --- | --- |
| [Advocate Aurora Research Institute](http://aurorahealthcare.org/) | Regional | OMOP | available |
| [Advocate Health Care Network](http://advocatehealth.com/) | CTSA | pending | pending |
| [Arkansas Children's Hospital](http://archildrens.org/) | CTSA | pending | pending |
| [Baylor College of Medicine](http://bcm.edu/) | Unaffiliated | pending | pending |
| [Boston University Medical Campus](http://bu.edu/) | CTSA | TRINETX | available |
| [Brown University](http://brown.edu/) | CTR | OMOP | available |
| [Carilion Clinic](http://carilionclinic.org/) | CTSA | TRINETX | available |
| [Charleston Area Medical Center](http://camc.org/) | Unaffiliated | TRINETX | available |
| [Children's Hospital Colorado](http://childrenscolorado.org/) | CTSA | OMOP | available |
| [Children's Hospital of Philadelphia](http://chop.edu/) | CTSA | pending | pending |
| [Children's National Hospital](http://childrensnational.org/) | CTSA | TRINETX | available |
| [Cincinnati Children's Hospital Medical Center](http://cincinnatichildrens.org/) | CTSA | pending | pending |
| [Columbia University Irving Medical Center](http://health.columbia.edu/) | CTSA | OMOP | available |
| [Duke University](http://duke.edu/) | CTSA | PCORNET | available |
| [Emory University](http://emory.edu/) | CTSA | pending | pending |
| [George Washington University](http://gwu.edu/) | CTSA | OMOP | available |
| [HonorHealth](http://honorhealth.com/) | Unaffiliated | pending | pending |
| [Icahn School of Medicine at Mount Sinai](http://mssm.edu/) | CTSA | OMOP | submitted |
| [Indiana University School of Medicine](http://regenstrief.org/) | CTSA | OMOP | available |
| [Johns Hopkins University](http://jhu.edu/) | CTSA | PCORNET | available |
| [Loyola Medicine](http://loyolamedicine.org/) | CTSA | PCORNET | available |
| [Loyola University Chicago](http://luc.edu/) | CTSA | pending | pending |
| [Maine Medical Center](http://mmcri.org/) | CTR | OMOP | available |
| [Massachusetts General Brigham](http://massgeneralbrigham.org/) | CTSA | ACT | available |
| [Mayo Clinic Rochester](http://mayo.edu/) | CTSA | ACT | submitted |
| [Medical College of Wisconsin](http://mcw.edu/) | CTSA | pending | pending |
| [Medical University of South Carolina](http://musc.edu/) | CTSA | ACT | available |
| [MedStar Health Research Institute](http://medstarhealth.org/) | CTSA | pending | pending |
| [MetroHealth](http://metrohealth.org/) | Unaffiliated | pending | pending |
| [Montana State University](http://montana.edu/) | CTR | pending | pending |
| [Montefiore Medical Center](http://montefiore.org/) | CTSA | OMOP | available |
| [Nemours](http://nemours.org/) | CTR | OMOP | available |
| [New York University Grossman School of Medicine](http://med.nyu.edu/) | CTSA | pending | pending |
| [NorthShore University Health System](http://northshore.org/) | CTSA | PCORnet | available |
| [Northwestern University at Chicago](http://northwestern.edu/) | CTSA | PCORNET | available |
| [OCHIN](http://ochin.org/) | Unaffiliated | PCORNET | available |
| [Ochsner Medical Center](http://ochsner.org/locations/ochsner-medical-center) | CTR | pending | pending |
| [Oregon Health & Science University](http://ohsu.edu/) | CTSA | OMOP | available |
| [Penn State](http://psu.edu/) | CTSA | TRINETX | available |
| [Rush University Medical Center](http://rush.edu/) | CTSA | PCORNET | available |
| [Rutgers, The State University of New Jersey](http://rutgers.edu/) | CTSA | OMOP | available |
| [Sanford Research](http://sanfordresearch.org/) | Unaffiliated | pending | pending |
| [Stanford University](http://stanford.edu/) | CTSA | pending | pending |
| [Stony Brook University](http://stonybrook.edu/) | Unaffiliated | TRINETX | available |
| [The Ohio State University](http://osu.edu/) | CTSA | PCORNET | available |
| [The Rockefeller University](http://rockefeller.edu/) | CTSA | pending | pending |
| [The Scripps Research Institute](http://scripps.edu/) | CTSA | pending | pending |
| [The State University of New York at Buffalo](http://buffalo.edu/) | CTSA | OMOP | available |
| [The University of Chicago](http://uchicago.edu/) | CTSA | PCORNET | available |
| [The University of Iowa](http://uiowa.edu/) | CTSA | PCORNET | available |
| [The University of Michigan at Ann Arbor](http://umich.edu/) | CTSA | PCORNET | available |
| [The University of Texas Health Science Center at Houston](http://uth.edu/) | CTSA | ACT | available |
| [The University of Texas Health Science Center at Tyler](http://uth.edu/) | CTSA | PCORnet | submitted |
| [The University of Texas Medical Branch at Galveston](http://utmb.edu/) | CTSA | TRINETX | available |
| [The University of Utah](http://utah.edu/) | CTSA | PCORNET | available |
| [Tufts Medical Center](http://tuftsmedicalcenter.org/) | CTSA | OMOP | available |
| [Tulane (University Medical Center New Orleans)](http://lphi.org/) | Unaffiliated | PCORnet | available |
| [University Medical Center New Orleans](http://umcno.org/) | CTR | PCORNET | available |
| [University of Alabama at Birmingham](http://uab.edu/) | CTSA | TRINETX | available |
| [University of Arkansas for Medical Sciences](http://uams.edu/) | CTSA | TRINETX | available |
| [University of California, Davis](http://ucdavis.edu/) | CTSA | OMOP | submitted |
| [University of California, Irvine](http://uci.edu/) | CTSA | OMOP | submitted |
| [University of California, Los Angeles](http://ucla.edu/) | CTSA | OMOP | submitted |
| [University of California, San Diego](http://ucsd.edu/) | CTSA | OMOP | submitted |
| [University of California, San Francisco](http://ucsf.edu/) | CTSA | OMOP | submitted |
| [University of Cincinnati](http://uc.edu/) | CTSA | TRINETX | available |
| [University of Colorado, Anschutz Medical Campus](http://cuanschutz.edu/) | CTSA | OMOP | available |
| [University of Florida](http://ufl.edu/) | CTSA | pending | pending |
| [University of Illinois at Chicago](http://uic.edu/) | CTSA | ACT | available |
| [University of Kansas Medical Center](http://kumc.edu/) | CTSA | PCORnet | available |
| [University of Kentucky](http://uky.edu/) | CTSA | ACT | available |
| [University of Massachusetts Medical School Worcester](http://umassmed.edu/) | CTSA | OMOP | submitted |
| [University of Miami](http://miami.edu/) | CTSA | PCORNET | available |
| [University of Minnesota](http://umn.edu/) | CTSA | ACT | available |
| [University of Mississippi Medical Center](http://umc.edu/) | CTR | OMOP | available |
| [University of Nebraska Medical Center](http://unmc.edu/) | CTR | PCORNET | available |
| [University of New Mexico Health Sciences Center](http://hsc.unm.edu/) | CTSA | pending | pending |
| [University of North Carolina at Chapel Hill](http://unc.edu/) | CTSA | PCORNET | available |
| [University of Oklahoma Health Sciences Center](http://ouhsc.edu/) | CTR | OMOP | available |
| [University of Rochester](http://rochester.edu/) | CTSA | OMOP | available |
| [University of Southern California](http://usc.edu/) | CTSA | TRINETX | available |
| [University of Texas Health Science Center at San Antonio](http://uthscsa.edu/) | CTSA | pending | pending |
| [University of Vermont](http://uvm.edu/) | CTR | TRINETX | available |
| [University of Virginia](http://virginia.edu/) | CTSA | OMOP | submitted |
| [University of Washington](http://washington.edu/) | CTSA | OMOP | available |
| [University of Wisconsin?Madison](http://wisc.edu/) | CTSA | TRINETX | available |
| [Vanderbilt University Medical Center](http://vumc.org/) | CTSA | OMOP | available |
| [Virginia Commonwealth University](http://vcu.edu/) | CTSA | ACT | available |
| [Wake Forest University Health Sciences](http://wakehealth.edu/) | CTSA | PCORNET | available |
| [Washington University in St. Louis](http://wustl.edu/) | CTSA | OMOP | available |
| [Weill Medical College of Cornell University](http://weill.cornell.edu/) | CTSA | OMOP | available |
| [West Virginia University](http://wvu.edu/) | CTR | TRINETX | available |
| [Yale New Haven Hospital](http://ynhh.org/) | CTSA | pending | pending |

**Supplemental References**

1. Haendel MA, Chute CG, Bennett TD, Eichmann DA, Guinney J, Kibbe WA, et al. The National COVID Cohort Collaborative (N3C): Rationale, design, infrastructure, and deployment. J Am Med Inform Assoc. 2021;28(3):427-43. doi: 10.1093/jamia/ocaa196. PubMed PMID: 32805036; PubMed Central PMCID: PMCPMC7454687.

2. Pfaff ER, Girvin AT, Gabriel DL, Kostka K, Morris M, Palchuk M, et al. Synergies between Centralized and Federated Approaches to Data Quality: A Report from the National COVID Cohort Collaborative. J Am Med Inform Assoc. 2021. Epub 20210930. doi: 10.1093/jamia/ocab217. PubMed PMID: 34590684; PubMed Central PMCID: PMCPMC8500110.

3. Bennett TD, Moffitt RA, Hajagos JG, Amor B, Anand A, Bissell MM, et al. Clinical Characterization and Prediction of Clinical Severity of SARS-CoV-2 Infection Among US Adults Using Data From the US National COVID Cohort Collaborative. JAMA Netw Open. 2021;4(7):e2116901. Epub 20210701. doi: 10.1001/jamanetworkopen.2021.16901. PubMed PMID: 34255046; PubMed Central PMCID: PMCPMC8278272.

4. Dixon BE, Wen C, French T, Williams JL, Duke JD, Grannis SJ. Extending an open-source tool to measure data quality: case report on Observational Health Data Science and Informatics (OHDSI). BMJ Health Care Inform. 2020;27(1). doi: 10.1136/bmjhci-2019-100054. PubMed PMID: 32229499; PubMed Central PMCID: PMCPMC7254131.

5. Visweswaran S, Becich MJ, D'Itri VS, Sendro ER, MacFadden D, Anderson NR, et al. Accrual to Clinical Trials (ACT): A Clinical and Translational Science Award Consortium Network. JAMIA Open. 2018;1(2):147-52. Epub 20180821. doi: 10.1093/jamiaopen/ooy033. PubMed PMID: 30474072; PubMed Central PMCID: PMCPMC6241502.

6. Topaloglu U, Palchuk MB. Using a Federated Network of Real-World Data to Optimize Clinical Trials Operations. JCO Clin Cancer Inform. 2018;2:1-10. doi: 10.1200/cci.17.00067. PubMed PMID: 30652541; PubMed Central PMCID: PMCPMC6816049.

7. Bian J, Lyu T, Loiacono A, Viramontes TM, Lipori G, Guo Y, et al. Assessing the practice of data quality evaluation in a national clinical data research network through a systematic scoping review in the era of real-world data. J Am Med Inform Assoc. 2020;27(12):1999-2010. doi: 10.1093/jamia/ocaa245. PubMed PMID: 33166397; PubMed Central PMCID: PMCPMC7727392.
